# High-resolution bioelectrical imaging of Aβ-induced network dysfunction on CMOS-MEAs for neurotoxicity and rescue studies

**DOI:** 10.1038/s41598-017-02635-x

**Published:** 2017-05-26

**Authors:** Hayder Amin, Thierry Nieus, Davide Lonardoni, Alessandro Maccione, Luca Berdondini

**Affiliations:** Nets3 Laboratory, Departement of Neuroscience & Brain Technologies (NBT), Fondazione Istituto Italiano di Tecnologia (IIT), via morego 30, 16163 Genova, Italy

## Abstract

Neurotoxicity and the accumulation of extracellular amyloid-beta_1–42_ (Aβ) peptides are associated with the development of Alzheimer’s disease (AD) and correlate with neuronal activity and network dysfunctions, ultimately leading to cellular death. However, research on neurodegenerative diseases is hampered by the paucity of reliable readouts and experimental models to study such functional decline from an early onset and to test rescue strategies within networks at cellular resolution. To overcome this important obstacle, we demonstrate a simple yet powerful *in vitro* AD model based on a rat hippocampal cell culture system that exploits large-scale neuronal recordings from 4096-electrodes on CMOS-chips for electrophysiological quantifications. This model allows us to monitor network activity changes at the cellular level and to uniquely uncover the early activity-dependent deterioration induced by Aβ-neurotoxicity. We also demonstrate the potential of this *in vitro* model to test a plausible hypothesis underlying the Aβ-neurotoxicity and to assay potential therapeutic approaches. Specifically, by quantifying N-methyl D-aspartate (NMDA) concentration-dependent effects in comparison with low-concentration allogenic-Aβ, we confirm the role of extrasynaptic-NMDA receptors activation that may contribute to Aβ-neurotoxicity. Finally, we assess the potential rescue of neural stem cells (NSCs) and of two pharmacotherapies, memantine and saffron, for reversing Aβ-neurotoxicity and rescuing network-wide firing.

## Introduction

Alzheimer’s disease (AD) is an irreversible, progressive, neurodegenerative brain ailment characterized by memory loss and synaptic dysfunction^1–3^. The accumulation of toxic soluble oligomers of extracellular amyloid-beta_1–42_ (Aβ) peptides plays a central causal role in the genesis of AD^4–6^ and is associated with the derangement of neuronal network activity, which is a common feature of AD as in other neurodegenerative disorders^7^. Notably, a growing body of evidence suggests that soluble Aβ-oligomers are more cytotoxic than fibrillar Aβ-aggregates and more potent in evoking various synaptic dysfunctions and abnormal neuronal activities^8–12^. However, the manner in which the toxicity of Aβ-oligomers predisposes and impairs neuronal network-wide activity before triggering neuronal death processes remains elusive.

In parallel, attempts to find cures for AD and neurodegenerative disorders are hampered both by the elusive causes of disease hallmarks and by the relative paucity of robust and sensitive assay systems for testing therapeutic strategies with the ability to restore misplaced neuronal network dynamics prior to cellular death. One increasingly common alternative strategy for confronting these challenges is to advance preclinical *in vitro* methods that possess complementary *in vivo* counterparts. This strategy enables us to probe-under early conditions of neurotoxicity onset the effects of insults associated with network functions, to pinpoint therapeutic targets, and to advance in testing potential neuroprotective agents prior to long and expensive clinical trials^13^.

One opportunity to advance preclinical methods involves taking advantage of recent, important progress in the application of cell culture systems and the development of microelectronic biosensing-chip technologies^14^. Notably, *in vitro* neuronal systems retain some fundamental functions underlying *in vivo* neurophysiological properties^15^ and provide better access for measurements and manipulations than *in vivo* animal studies. In particular, combined with emerging large-scale electrical recording technologies, neuronal cultures can lead to *in vitro* cell-based platforms that may allow the implementation of disease-specific models for studying basic mechanisms of neurodegeneration within networks and for the parallel testing of various potential pharmacological agents or cell-based therapies.

Among the different techniques for measuring bioelectrical activity in cellular networks, conventional extracellular *in vitro* recording methods, such as multielectrode arrays (MEAs), provide multisite, label-free, non-invasive and long-term monitoring of spontaneous spiking activity in neuronal cultures; hence, they enable both the detection of the induced toxicity responses of neuronal networks and characterization of the effect of potential therapeutics^16–20^. However, conventional MEAs typically integrate a few tens up to a few hundreds of recording sites; such a low spatial resolution down-samples the network-wide extracellular spiking activity. As we have previously shown, this shortcoming can affect the estimation of mean activity parameters^21^. Additionally, because of the low spatial resolution of conventional MEAs, these devices preclude the electrical characterization of the network dynamics at the cellular level. Functional optical imaging such as Ca^2+^ imaging can perform multiple single-cell activity mapping but is typically limited by the low temporal resolution and by the difficulty in conducting chronic ongoing recordings over several days^22^.

To overcome these limitations and to allow network-wide activity to be followed for extended time periods, in this study, we exploited the high spatiotemporal bioelectrical imaging performances of complementary metal-oxide semiconductor-multi-electrode arrays (CMOS-MEAs)^23–25^. These devices simultaneously record from several thousands of single neurons in cultured networks^23^ and may reveal high-information-content network responses available only from the interplay of different components of neuronal ensembles^26^. By exploiting this chip technology and optical readouts, we thoroughly characterize the early activity-dependent changes induced by toxic Aβ-oligomers in neuronal networks. We show that large-scale electrical recordings enable the induced activity changes of nanomolar concentrations of Aβ-oligomers to be monitored by enabling the quantification of mean activity parameters that show a very low inter-culture variability. Additionally, the ability to monitor a large number of neurons simultaneously allows researchers to follow end-point changes in the lognormal-like firing frequency distribution^27, 28^ of single neurons that comprise each cultured network. Furthermore, we demonstrate the potential of this *in vitro* AD model for use in toxicity and rescue studies. In particular, a potential and recognized cellular target underlying Aβ-induced neurotoxicity associated with AD involves the activation of N-methyl D-aspartate (NMDA) receptors (NMDARs) that has been suggested to underlie such deterioration.

Here, we show that our *in vitro* model allows us to identify an NMDA concentration that mirrors the network activity deregulation induced by Aβ-toxicity, revealing and confirming at the network level a critical role of extrasynaptic NMDARs activation. Finally, we determine the effects of various potential neuroprotective strategies to reverse Aβ-toxicity, including memantine, which is a clinically applied NMDARs antagonist, and we explore the effects of a potential natural compound (saffron) and a cell-based therapeutic approach using NSCs (adult neural stem cells).

## Results

### High-resolution electrical readouts reveal activity-dependent changes induced by neurotoxic Aβ-oligomers

**Figure 1:**
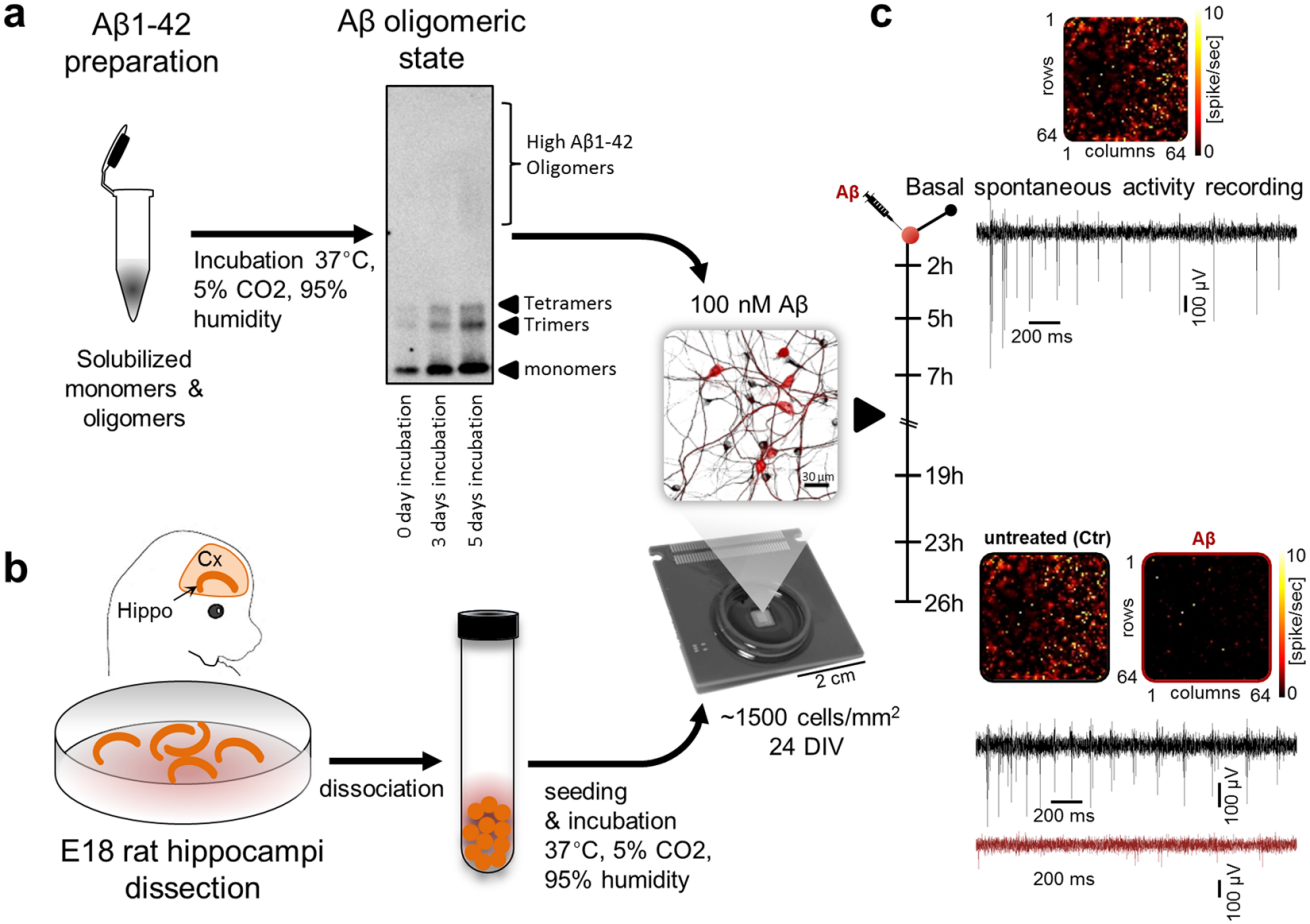
Schematic illustration of the experimental neurodegeneration model on-CMOS-MEA chips. (**a**) Preparation and optimization of Aβ-oligomers. (**b**) Preparation and seeding of embryonic hippocampal neurons on CMOS chips. (**c**) Monitoring the spontaneous firing activity from 4096 electrodes on CMOS chips at baseline and after 0.1 µM Aβ-oligomer treatment.

**Figure 2:**
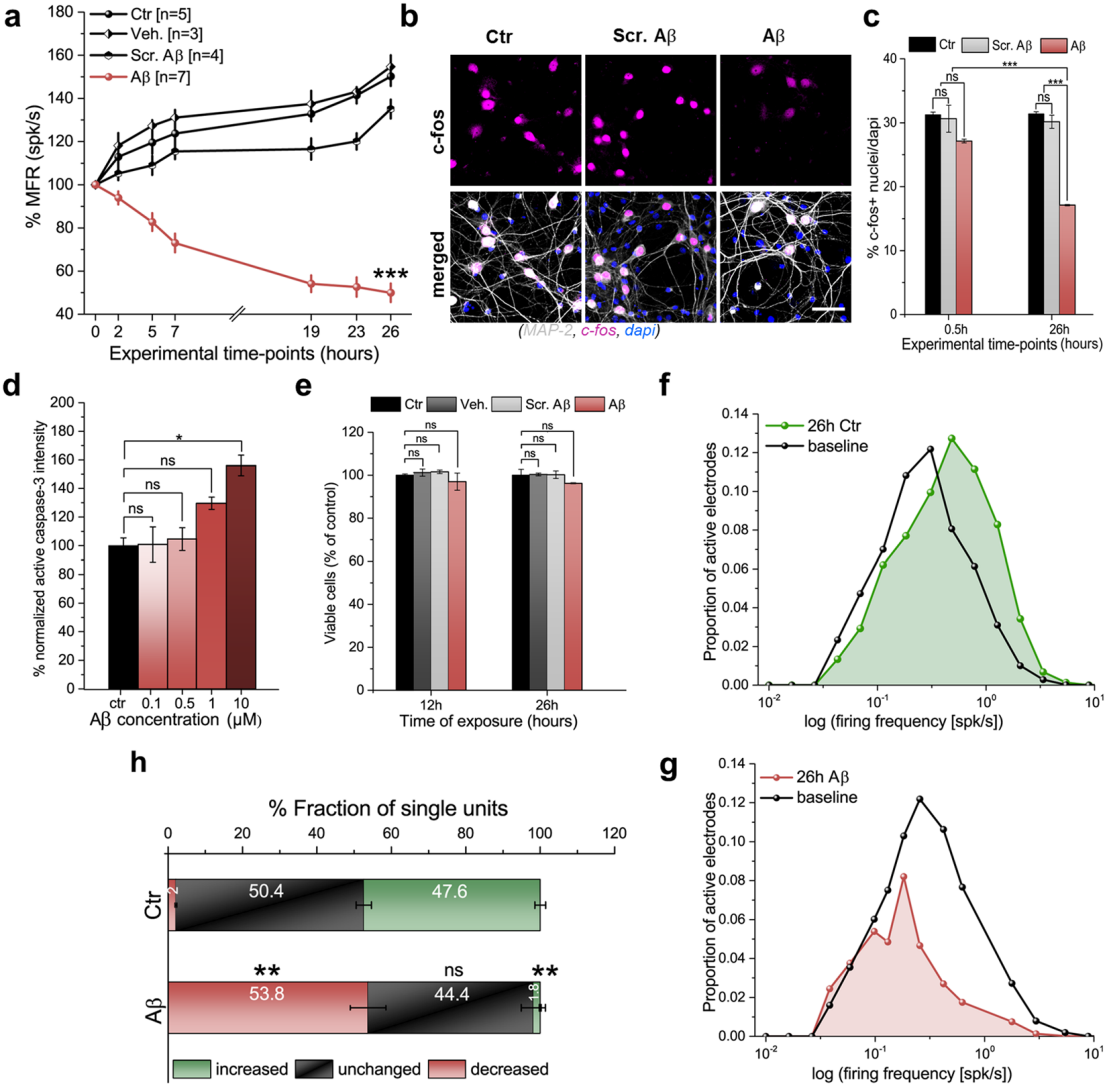
Quantification of activity-dependent changes in neuronal network induced by early Aβ-neurotoxicity. (**a**) Large-scale array electrical readouts (MFR) of control groups (black lines) versus the 0.1 μM Aβ-induced toxicity group (red line). ***p < 0.001, ANOVA. (**b**) Confocal micrographs showing cellular c-fos expression after 26 h, cross-validated at the cellular level the electrical readouts. Scale bar represents 50 μm. (**c**) Quantification of c-fos^+^ nuclei ratio shows a significant decrease in expression 26 h after Aβ-exposure (red) compared to that in control groups (black and gray). ***p < 0.001, ANOVA. At the considered time points, the c-fos and electrical readouts show similar significant changes. (**d**) Intensity quantification of caspase-3 activity 26 h after Aβ-increased concentration shows no apoptotic activation at 0.1 μM, whereas such activation is significantly present at 10 µM. *p < 0.05, ANOVA. (**e**) Quantification of MTT assay shows no significant cell death at 12 and 26 h after 0.1 μM Aβ-exposure. (**f**) Distributions of firing rates (baseline and after 26 h) for the untreated condition, showing a significant peak shift toward higher frequencies of the preserved lognormal-like distributions, *p* < 0.05, Kolmogorov-Smirnov test. (**g**) Distributions of firing rates (baseline and after 26 h) for 0.1 μM Aβ-exposure, showing a significant peak shift toward lower frequencies and loss of the lognormal-like distribution, *p* < 0.05, Kolmogorov-Smirnov test. (**h**) Quantification of single active units showing increased, decreased or unchanged fractions of firing rates after 26 h, with respect to baseline, for untreated and Aβ-treated cultures. **p < 0.01, ANOVA.

We developed a cell culture model of Alzheimer’s disease (AD) on CMOS-MEAs (Fig. 1). Rat hippocampal neurons were grown on-chip with an optimized surface functionalization consisting of poly-DL-ornithine (PDLO) as a biochemical substrate. To mimic AD pathogenesis, neurotoxicity was induced under controlled conditions with a synthesized Aβ_(1–42)_ peptide that was used to prepare and optimize the precise oligomeric state (tetramers). At 24 days *in vitro* (DIV), control and Aβ-oligomers solutions were added to the cell culture media after recording the baseline activity of each sample with the 4096 electrodes of the CMOS-MEAs. Then, we recorded the extracellular neuronal spiking activity at seven time points, from baseline until 26 h, both from three cell culture control groups (untreated, scrambled amyloid-β and vehicle) and from cultures treated with a low concentration (0.1 µM) of Aβ-oligomers. All cultures displayed reproducible baseline network-wide spontaneous spiking activity, while samples treated with Aβ-oligomers showed a progressive network dysfunction (Supplementary Fig. S1). To monitor these network-wide responses, we first computed different mean activity parameters. The Aβ-treated group showed a prominent decrease in the mean firing and bursting activities and in the number of firing neurons (Fig. 2a and Supplementary Fig. S2a,b), while after 26 h, controls maintained their level of activity. Remarkably, with these large-scale electrode array recordings and optimized cell culture conditions, we achieved a very low inter-culture and inter-animal variability and a high statistical significance of the mean activity parameters computed for each group of cell cultures.

To evaluate the role of the electrode density on the fidelity of these readouts, we performed the same analysis by scaling down the electrode density of the same data to a similar layout as conventional 60-electrode arrays. As shown in Supplementary Fig. S2c–e, the quantification of the mean firing activity of the down-sampled network showed significantly different mean values and a high variability. This condition is attributable to the insufficient resolution of the low-density electrode array layout that provided an erroneous and skewed interpretation of firing activity changes elicited by Aβ-oligomers. Additionally, variations in the number of active neurons cannot be discerned (Supplementary Fig. 2e). To confirm that the disrupted network-wide activity recorded with CMOS-MEAs originated from cellular dysfunction, we assayed cellular neuronal activity using c-fos immunofluorescence upon Aβ-exposure (Fig. 2b). We observed a decrease in the number of c-fos-positive nuclei in cultures treated with Aβ for 26 h but no change in the c-fos expression of control groups (Fig. 2b,c). The c-fos readout was confirmed by a positive control group treated with 30 mM of potassium chloride, showing a stable increase in the c-fos expression over 26 h (Supplementary Fig. S2f,g).

Given that neuronal cell death associated with Aβ-induced neurotoxicity might occur via multiple pathways^29, 30^, we investigated whether the induced dysregulated responses of cellular and network-wide activities were caused by neuronal inviability. To this end, we assayed apoptotic cellular death upon 0.1 µM Aβ-exposure by using caspase-3 immunofluorescence and the MTT-colorimetric method. We observed no significant caspase-3 expression after 26 h and 48 h (Fig. 2d and Supplementary Fig. S3a,b). Rather, cellular death was observed earlier both for higher concentrations of Aβ (after 26 h at 10 µM, Fig. 2d and Supplementary Fig. S3a,b) and for longer exposure time (after 48 h at 1 µM, Supplementary Fig. S3a,b). Consistently, MTT readouts showed no significant cellular death at 0.1 µM of Aβ-oligomers up to 72 h of exposure (Fig. 2e and Supplementary Fig. S3c). Therefore, the loss of the cellular and spontaneous network activities observed with electrical and c-fos readouts was an early response that occurred before cellular death and was associated with the toxicity of 0.1 µM Aβ-oligomers. To evaluate whether 0.1 µM Aβ-oligomers induced a functional reorganization of the network, we took advantage of our large-scale recordings to quantify changes in the lognormal-like distribution, as previously reported for *in vivo* ^31^ and *in vitro* ^27^ conditions. We found that after 26 h, the lognormal-like distribution was skewed toward high frequencies in the control group (Fig. 2f) and low firing frequencies in the Aβ-treated group (Fig. 2g). We next investigated whether this derangement of the population firing frequencies arose from changes in the firing of single neuronal units (see the **Methods** section for extracellular recordings and analyses of spiking activity). Such changes were found by quantifying fractions of significant changes in the distributions of the inter-spike interval (ISI) of the spiking activity for each unit and by comparing baseline versus 26 h recordings of control and Aβ groups (Supplementary Fig. S4a,b). In Aβ-treated cultures, a large fraction of single units, 53.8 ± 4.7%, reduced their firing activity, while only 1.8 ± 0.2% increased their activity, which is significantly different from the fractions obtained from the control group (Fig. 2h).

### Concentration-dependent interplay between Aβ-excitotoxicity and the activation of extrasynaptic NMDARs

**Figure 3:**
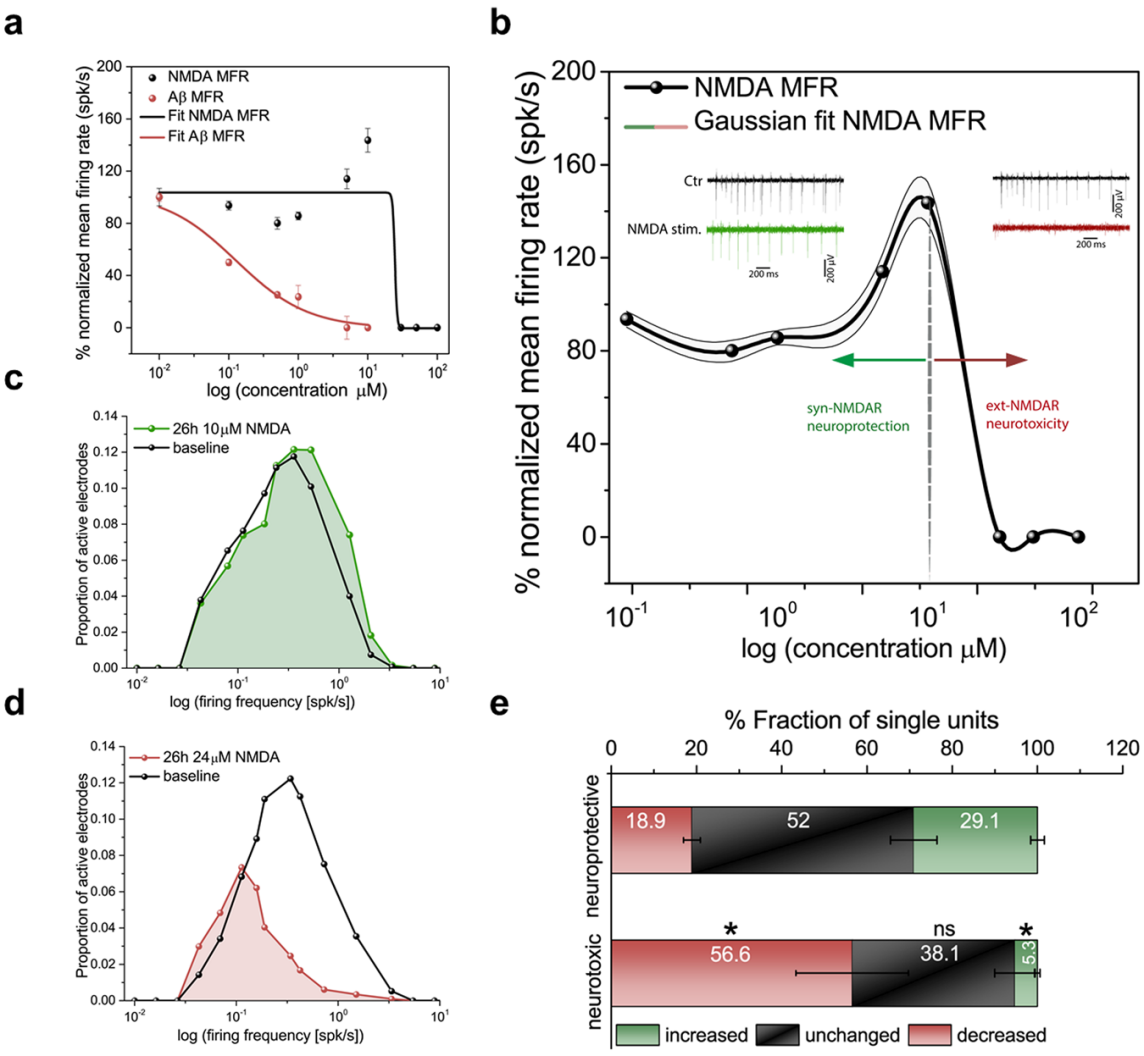
Testing the NMDAR target mechanism hypothesis to emulate early Aβ-neurotoxicity. (**a**) Concentration-response curve of MFR for (0.1, 0.3, 0.5, and 1 µM) Aβ and (0.1, 0.5, 1, 5, 10, 30, 50, and 100 µM) NMDA after 26 h treatment. The goodness R^2^ values of these fit curves are 0.98 for Aβ-data, and 0.89 for NMDA-data, respectively. (**b**) Arrows from the borderline that is indicated at 10 µM NMDA on the normalized MFR distinguish between effects of low NMDA doses (left, green) responsible for neuroprotection and high NMDA doses (right, red) responsible for neurotoxicity. (Insets: Spontaneous extracellular signal traces before and after 26 h of NMDA treatment (left) with 10 µM and (right) with 24.5 µM. (**c**) Distribution of firing rates after 26 h of exposure to a neuroprotective NMDA dose (10 µM) shows a similar lognormal-like distribution to baseline, with a slight non-significant peak shift toward higher frequencies, *p* = 0.22, Kolmogorov-Smirnov test. (**d**) At 24.5 µM NMDA, i.e., a concentration reported in this study to link Aβ excitotoxicity and extrasynaptic NMDARs activation, the firing distribution shows abnormal network dynamics, loss of the lognormal-like distribution and a peak shifted toward low rates, *p* < 0.05, Kolmogorov-Smirnov test. (**e**) Quantification of single active units showing increased, decreased or unchanged fractions of their firing rates after 26 h with respect to baseline, for cultures treated with a neuroprotective dose (10 µM) and a neurotoxic dose (24.5 µM) of NMDA. *p < 0.05, ANOVA.

Evidence suggests that alteration in the activity of NMDA receptors (NMDARs) plays a key role in Aβ-induced neurotoxicity associated with AD, making these receptors major drug targets for AD therapies^10, 32, 33^. To demonstrate our *in vitro* AD model for testing our hypothesis of Aβ-neurotoxicity, we thus asked whether it is possible to identify a specific concentration of NMDA agonist that could block specific NMDARs to give rise to a very similar electrical phenotype as that observed with 0.1 µM Aβ-oligomers. To address this question, we conducted CMOS-MEA recordings to quantify the concentration-dependent effects of Aβ-oligomers and of NMDA (Fig. 3). When the Aβ-oligomer concentration reached 1 µM, the mean firing rate decreased to 23.49 ± 2.31% from the baseline measured activity, while the activity was abolished for a concentration of 10 µM (Fig. 3a, red line). With NMDA, the analysis of the induced concentration-dependent effects showed a more complex response than those induced by Aβ-oligomers (Fig. 3a, black line). Indeed, after 26 h, at 1 µM of NMDA, the network activity was characterized by a slight decrease of the mean firing rate. For concentrations between 5 µM and 10 µM of NMDA, we observed a significant increase in activity. However, at 30 µM and 50 µM of NMDA, the mean firing rate rapidly decreased, and the spiking activity was completely abolished at 100 µM of NMDA. The IC_50_ values for both Aβ-oligomers and NMDA were computed by fitting the non-linear dose-response curves using the Hill equation and yielded 24.576 ± 2.9% µM for NMDA and in 0.12 ± 0.09% µM for Aβ-oligomers (very close to the low concentration of Aβ-oligomers used to induce dysfunction in our model). The goodness of these fits was evaluated using the adjusted R^2^ parameter, which yielded 0.98, and 0.89 for Aβ-oligomer and NMDA data, respectively.

These concentration-dependent effects of NMDA are in accordance with the reported effects of synaptic versus extrasynaptic NMDARs activation^34^. As we observed, this implies that a low dose of NMDA (10 µM) induces synaptic NMDARs activation, promotes neuroprotection and increases network activity. In contrast, a high dose of NMDA (24.576 ± 2.9% µM) instigates neurotoxicity, suppresses firing rates, and activates extrasynaptic NMDARs^35^. To better appreciate our data showing that those NMDA concentrations >20 µM do activate neurotoxicity signals, arrows are used to delineate two distinct neuroprotective and neurotoxic regions induced by the concentration-dependent effects of NMDA (Fig. 3b). This approach allowed us to identify an NMDA concentration of 24.5 µM that through the overactivation of extrasynaptic NMDARs, induced a disruption of the network-wide electrical activity similar to the effects induced by 0.1 µM Aβ-oligomers. These data are in accordance with previously reported data indicating that the overactivation of extrasynaptic NMDARs is a key mechanism underlying the electrical phenotype induced by Aβ-oligomers. Central to these results are reports on the role of the prolonged activation of extrasynaptic pools of NMDARs as the actual factor inducing an increase in neuronal Aβ production^36^.

To further confirm that the observed global firing was correlated with neuroprotective effects at 10 µM NMDA and neurotoxic effects at 24.5 µM NMDA, we analyzed the distribution of the multiunit neuronal firing frequencies for these specific recorded groups (Fig. 3c,d). Consistent with our previous results on 0.1 µM Aβ-oligomers (Fig. 2f,g), we observed that after 26 h, the lognormal-like distribution was skewed toward high frequencies for 10 µM NMDA (Fig. 3c), while it was skewed toward low frequencies for cultures treated with 24.5 µM NMDA (Fig. 3d). Notably, the distribution observed in the NMDA neurotoxic group differed from that obtained for Aβ-treated cultures. This difference indicates the establishment of different network dynamics, likely denoting the Aβ neurotoxic activation of parallel pathways to the overactivation of extrasynaptic NMDARs. Additionally, the analysis of the individual firing activity revealed that a large fraction of units in the neurotoxic activated group (56.5 ± 13.2%) reduced their firing activity, while only 5.3 ± 0.5% showed an increase from their pre-treatment baseline. This finding is significantly different from the neuroprotective group but similar to what we observed with Aβ-oligomers (Fig. 3e and Supplementary Fig. S5a,b).

### The effects of pharmacotherapies (memantine and saffron) for neuroprotection and reversing Aβ-toxicity in neuronal networks

**Figure 4:**
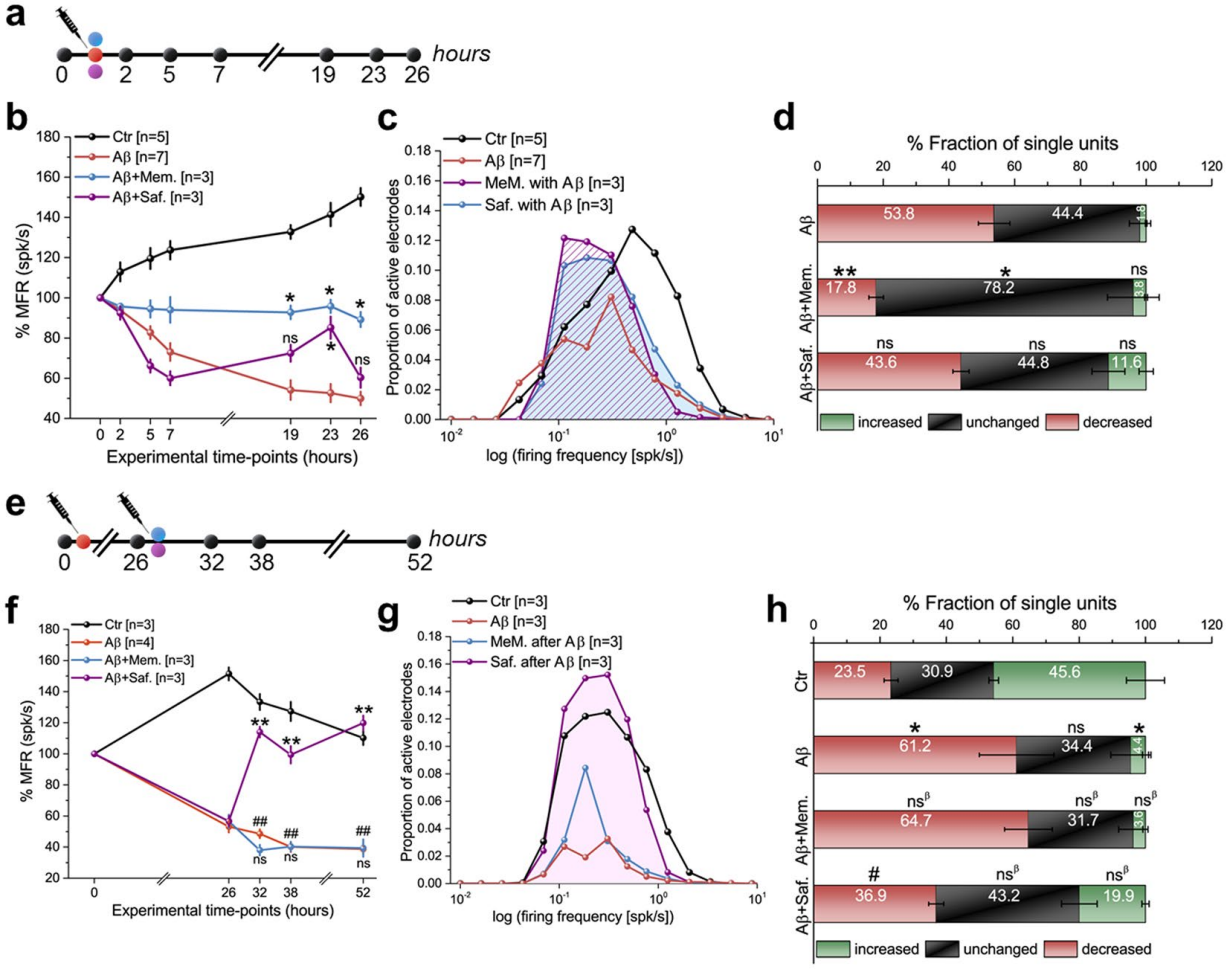
Rescue strategies using biochemical molecules (memantine) and (saffron) to neuroprotect and reverse Aβ-induced neurotoxicity on CMOS chips. (**a**) Schematic of the 1^st^ scenario of rescue therapies using 10 µM memantine and 25 µg/ml saffron co-administered with 0.1 µM Aβ. (**b**) MFR responses of the 1^st^ scenario rescue therapy. *Denotes *p* < 0.05 compared to Aβ, ANOVA. ns, not significant. (**c**) Lognormal-like distributions of firing frequencies after 26 h in control and treated groups. *p* < 0.05, Kolmogorov-Smirnov test for biochemical treatments (memantine and saffron) versus Aβ. (**d**) Quantification of single active units. **Denotes *p* < 0.01 compared to Aβ, ANOVA. ns, not significant. (**e**) Schematic of 2^nd^ scenario rescue therapies, as in (a) but with molecules administered 26 h after 0.1 µM Aβ-oligomers and responses monitored for 52 h. **(f)** MFR responses of the 2^nd^ rescue strategy. **Denotes *p* < 0.01 compared to Aβ, ^##^denotes *p* < 0.01 compared to control group, ANOVA. (**g**) As in (**c**) but corresponding to treatment in (**e**). *p* < 0.05 and *p* = 0.18, Kolmogorov-Smirnov test for saffron versus 0.1 µM Aβ and memantine versus 0.1 µM Aβ, respectively. (**h**) Quantification of single units as in (**d**) but for the 2^nd^ scenario treatment. *Denotes *p* < 0.05 compared to control, ^#^denotes *p* < 0.05 compared to Aβ, ANOVA. ns and ns^β^ denote not significant compared to control and Aβ, respectively.

To demonstrate the potential of the proposed *in vitro* AD model for drug development, we tested the efficacy of two pharmacotherapies by applying 10 µM memantine and 25 µg/ml saffron. These experiments were performed under two experimental scenarios, with and without pre-established neurodegeneration (Fig. 4a,e).

Memantine is a clinically used drug in human AD therapy that acts as an antagonist of NMDAR-mediated glutamate excitotoxicity at both single receptor and synaptic levels. This drug provides neuroprotection with minimal adverse effects by interacting with targets only during pathological insult and not during physiological conditions^37^. Several mechanisms have been suggested to explain the efficacy of memantine in the treatment of neurodegenerative disorders. In our study, we used this drug because it is particularly effective in blocking excessive extrasynaptic NMDARs activity^38^ and consequently halts Aβ-induced toxicity^36, 39^. *Crocus sativus* L., commonly known as the saffron crocus, is an emerging new potential therapeutic compound for various neurodegenerative disorders, including AD. It has been reported that the antagonizing effect of crocin, i.e., a single-carotenoid molecule among the various molecules that comprise saffron (the dried stigmas of the saffron crocus), rescued the loss of learning and memory induced by ethanol^40^, enhanced the cognitive function of mild to moderate AD subjects^41^, and inhibited the aggregation and deposition of Aβ-fibrils^42^.

In the first pharmacological scenario, when memantine or saffron were co-administered with Aβ-oligomers (Fig. 4a), memantine displayed significant efficacy in reversing the Aβ-oligomer-induced neurotoxicity. In contrast, saffron showed an inconclusive effect (Fig. 4b and Supplementary Fig. S6). By investigating the effects of these compounds in recovering the lognormal-like firing frequency distribution, we found that although the distributions of memantine and saffron groups were skewed toward low frequencies after 26 h, the lognormal-like distributions were preserved and showed higher peaks than the Aβ-treated group (Fig. 4c). Quantification of single-unit spiking activity changes according to their ISI distributions showed that only memantine significantly maintained this feature. In this case, the fraction of single units decreased their firing rate in memantine-treated cultures (17.8 ± 2.2%) compared to that in Aβ-oligomer-treated cultures (53.8 ± 4.7%) (Fig. 4d and Supplementary Fig. S9). In the second scenario, when compounds were administered 26 h after Aβ-oligomers, the monitoring of the responses over 52 h (Fig. 4e) showed that saffron remarkably rescued the network activity and protected the cultures, while no significant effect was observed with memantine (Fig. 4f and Supplementary Fig. S7a,b). Interestingly, saffron also preserved the lognormal-like distribution after 52 h of Aβ-insult (Fig. 4g). This effect was also confirmed by the quantification of single-unit spiking activity changes according to their ISI distributions, showing that saffron-treated cultures displayed a significantly lower fraction of single units that decreased their firing rate (36.9 ± 2.2%) than Aβ-oligomer-treated cultures (61.1 ± 11.2%) (Fig. 4h and Supplementary Fig. S10).

### Studying the cell-therapeutic effect of adult neural stem cells for reversing Aβ-toxicity in neuronal networks

**Figure 5:**
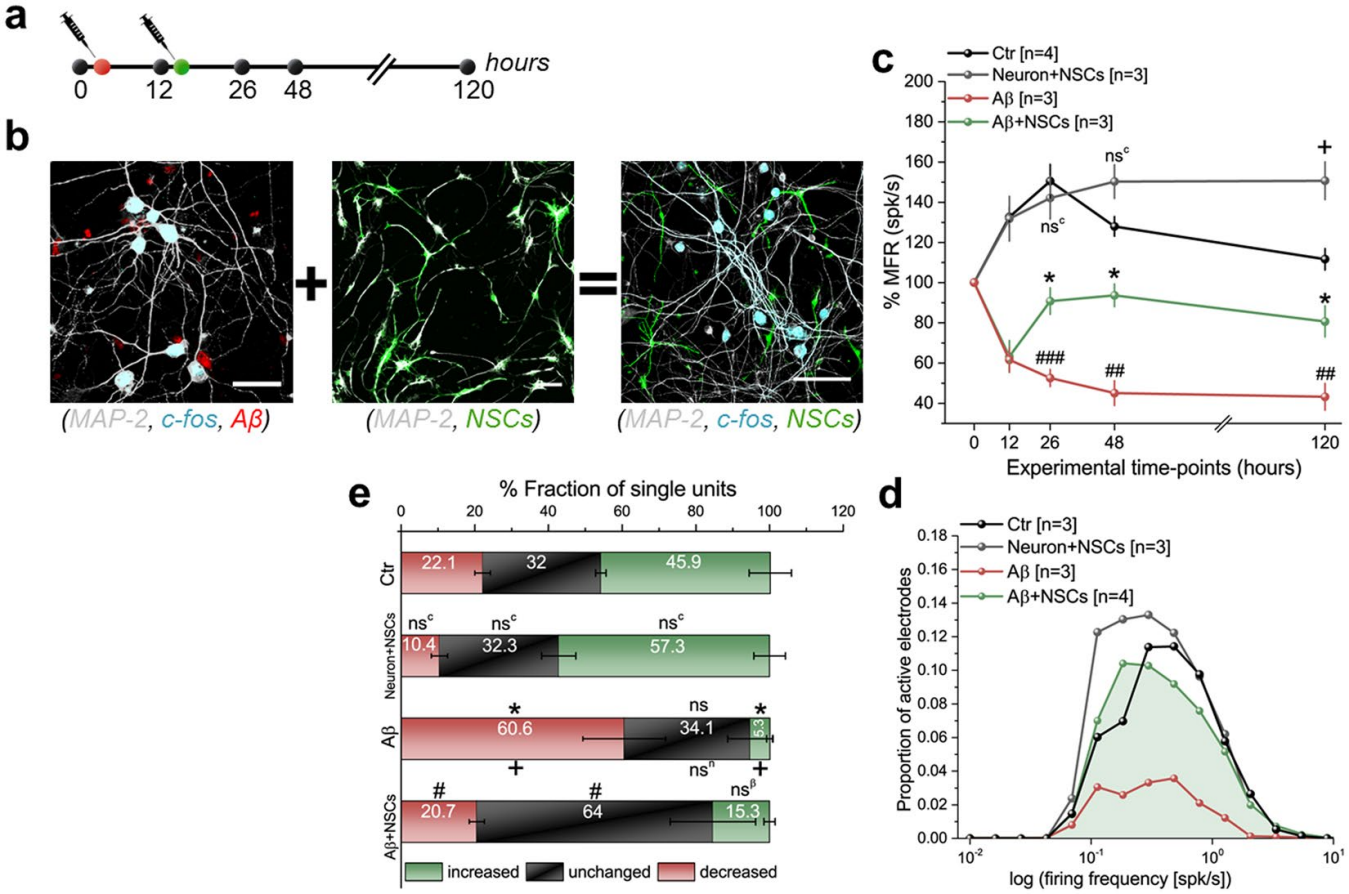
Cell-based therapeutic strategy using neural stem cells (NSCs) to reverse Aβ-induced neurotoxicity on CMOS chips. (**a**) Schematic of therapeutic treatment using NSCs administration on-chip 12 h after 0.1 µM Aβ. (**b**) Confocal micrographs of diseased neuronal culture (*left*), network of NSCs (*middle*), and mixed populations of matured neurons and NSCs (*right*). Scale bars represent 50 μm. (**c**) On-chip MFR upon rescuing therapy using NSCs monitored for 120 h. *Denotes *p* < 0.05 compared to Aβ; ^#^ and ^+^denote *p* < 0.05 compared to control, ANOVA. ns^c^ denotes not significant compared to control. (**d**) Lognormal-like distributions after 48 h recording corresponding to rescuing strategy in (**a**). *p* < 0.05, Kolmogorov-Smirnov test for rescued networks (Aβ + NSC) versus diseased networks (Aβ). (**e**) Quantification of single-unit analysis for NSCs rescue strategy, * and ^+^denote *p* < 0.05 compared to control and Neuron + NSCs, respectively. ^#^Denotes *p* < 0.05 compared to Aβ, ANOVA. ns, ns^β^, and ns^c^ denote not significant compared to control, Neuron + NSCs, and Aβ, respectively.

Adult neural stem cells (NSCs) prolong both the self-renewal capacity and the ability to differentiate into multiple specialized cellular lineages^43^, suggesting radical new potential therapies for neurodegenerative disorders by providing neuroprotection either by enhancing trophic factors or by alleviating neurotoxic levels in diseased tissues^44, 45^. Here, to complement our demonstration of the use of our *in vitro* AD model for assays on pharmacological compounds, we also tested the progeny of NSCs for rescuing a network from Aβ-induced functional neurotoxicity (Fig. 5a). To this end, NSCs derived from adult hippocampal neurons were seeded and cultured on a pre-existing neuronal network that was exposed to Aβ-oligomers 12 h before adding the NSCs (Aβ + NSCs, Fig. 5b). After 14 h from the addition of NSCs, we observed a rapid recovery of the network-wide activity. Interestingly, this significant rescue of the mean firing rate, number of active electrodes and mean bursting rate was preserved for a long time (over 120 h) (Fig. 5c and Supplementary Fig. S8). The analyses of the lognormal-like distribution and ISI distributions of single-unit spiking activity changes, performed on data obtained 48 h after the baseline recordings, showed that the Aβ + NSCs cultures resulted in a drastic enhancement in the network dynamic by amending the altered network distributions observed in Aβ-cultures, leading to distributions similar to those of control cultures (Fig. 5d). The rescue of the population dynamic was also confirmed at the level of individually firing neurons, as indicated in Aβ + NSCs cultures by a lower fraction of single units (20.7 ± 2%) that showed decreased firing activity and a higher unchanged fraction (64 ± 11.5%) than in Aβ-treated cultures (Fig. 5e and Supplementary Fig. S11). Interestingly, these results at 48 h after the addition of NSCs were further confirmed for recordings at 120 h after Aβ-treatment (Supplementary Fig. S12).

## Discussion

The lack of reliable and robust quantifications of network responses to disease-associated neurotoxic insults is a key factor that limits the understanding and treatment of neurodegenerative disorders. In our study on Aβ-neurotoxicity, we demonstrated an *in vitro* methodology based on high-resolution CMOS-MEA recordings and optimized cell culture conditions for non-invasive, long-term electrical monitoring of neurodegenerative network activity changes with single-unit resolution. This approach also enables the study of neurotoxicity underlying the functional network-wide derangements and the true effects of potential anti-neurodegenerative drugs and neurogenic therapies.

Our results demonstrate that with the proposed methodology, we have reached a sensitivity that allows us to monitor network activity changes for nanomolar concentrations of Aβ-oligomers over 26 h at an early time of the neurodegeneration onset and, importantly, without cellular death over the experimental time window. This Aβ-oligomer concentration is plausible based on previously reported pathophysiological conditions^46^. Additionally, we show that this *in vitro* AD model allows us both to correlate Aβ-toxicity with previously reported NMDAR-dependent excitotoxicity^35^ and to test rescue strategies under conditions of early toxicity prior to cellular loss. These sensitivity performances of our electrical readouts for the effects induced by nanomolar Aβ-oligomer concentrations were not achieved in previous studies^19^ that used conventional 60-MEAs and required much higher lethal micromolar concentrations of Aβ-oligomers, which our study found to be associated with cellular loss.

Our electrical measures were cross-validated with quantifications of c-fos expression at specific time points. Optical readouts of c-fos expression have been described^47, 48^ as a tool to provide cellular resolution for studying the function of neuronal activity. We show that our electrical readouts match those based on cellular c-fos expression, only with the advantage of array-based electrical measures for monitoring changes in the firing dynamics. Uniquely, in addition to a very low inter-culture variability when estimating mean activity parameters, large-scale neuronal recordings allow us to follow the dynamic of the lognormal-like distribution of the single-unit firing frequencies within the entire network. Because of the low number of spiking neurons after 26 h from the Aβ-exposure (active electrodes reduced by at least 40%, Supplementary Fig. S2a), the use of conventional 60-MEAs would miss the estimation of such distribution until the end point. Interestingly, upon Aβ-induced toxicity this analysis of high-resolution recordings reveals a derangement of the network dynamics with a shift from the right (fast-firing minority of neurons) to the left (low-firing majority of neurons). This effect might be the consequence of larger changes in the firing of neurons that constitute the fast-firing minority population, which primarily comprise the highly active subnetwork or “hub” regions^28^, than those in their opponents identified at basal conditions before Aβ-exposure. In accordance with this interpretation of our results, the evidence suggests that highly connected “hub” regions are particularly prone to the attack of Aβ deposition and AD progression^49^. This latter result shows the potential of our approach to identify neuronal populations in which the Aβ toxicity begins and propagates within the network.

In a second series of experiments, we demonstrated the potential to exploit our *in vitro* AD model to study Aβ-neurotoxicity. Several studies strongly support the proposition that alterations in the activity of NMDARs play a key role in Aβ-induced toxicity associated with AD^10, 50, 51^. Thus, as a case study, we tested whether the activation of specific NMDARs might reproduce the network dysfunctional phenotype induced by Aβ-neurotoxicity. Our results show a detrimental concentration-dependent activation of extrasynaptic NMDARs at 24.5 μM of NMDA that gives rise to similar activity-dependent changes observed with 0.1 μM of Aβ-oligomers. Memantine at low micromolar concentrations is considered the only known therapeutically tolerated NMDA antagonist that preferentially blocks extrasynaptic over synaptic NMDARs activation^38^ and consequently halts Aβ-induced toxicity^36, 39^. Our data on neuroprotection obtained using 10 µM concentration of memantine co-administered with Aβ-oligomers are in accordance with the reported effects of memantine^36^. In turn, the reported concentration of NMDA might be used to implement a screening method for testing new anti-Aβ toxicity molecules targeting the abnormal activation of NMDARs.

In addition to memantine, we further demonstrated the use of our *in vitro* AD model for pharmacological rescue studies by testing the effects of a natural compound. The evidence suggested the neuroprotective effects of saffron in neurodegeneration^40–42^, although its effects and mechanisms of action on the activity of neuronal networks had not been revealed. Therefore, we evaluated whether saffron has neuroprotective potential for reversing *in vitro* network dysfunctions distinctively both during and after neurotoxicity that was elicited by 0.1 µM of Aβ-oligomers. Whereas saffron did not show clear effects in the first condition, we observed a remarkable neuroprotective capability of this complex molecule in the second condition. Overall, these results demonstrate the capability of our *in vitro* assays for testing the neuroprotective potential of complex natural compounds under different conditions of induced neurotoxicity. Additionally, our data might open up a new avenue to study the mechanisms underlying the neuroprotective effects of saffron for future therapeutic developments.

Finally, we also demonstrated the use of our *in vitro* AD model for testing a neurogenic therapy. Adult neuronal stem cells (NSCs) exhibit therapeutic efficacy in neurodegenerative disorders by acting through multiple and still unclear mechanisms to alleviate neurotoxic levels in disease conditions^44, 45^. Taking this evidence into account, we assessed the potential therapeutic effect of NSCs in our *in vitro* AD model by adding NSCs to a pre-existing hippocampal network previously incubated for 12 h with 0.1 µM Aβ-oligomers. Intriguingly, our results showed recovery of the network spiking and bursting activity (Fig. 5c and Supplementary Fig. S8b). Notably, these findings are in accordance with previous observations indicating that Aβ-accumulation was insufficient to impair the NSC population in APP mouse models^52^, whereas NSCs exhibiting immature GABAergic neuronal mechanisms resisted neurodegeneration in AD pathogenesis^53^. Given NSCs’ capability to reverse toxicity shown in our results, is tempting to speculate that these new added NSCs networks might have functional properties that assist in creating a surrogate for the dysfunctional existing network induced by the toxicity of Aβ-oligomers^54^. Important to note that the characterization of the intrinsic firing activity of the NSC networks alone is beyond the scope of the current work and it has previously been reported^55, 56^.

Overall, in this work, we demonstrate the potential of our *in vitro* AD model for studying the neurotoxicity of Aβ-oligomers at early onset and assess the potential of neuroprotective rescue strategies under different conditions by exploiting the recording capabilities of high-resolution MEAs and optimized cell culture conditions. In the near future, the presented approach might be extended toward scalable parallel readouts from multi-well CMOS-based electrode array formats to networks of human-derived neurons grown on CMOS-MEAs^27^. This application would allow us to study the effects of different disease-associated neurotoxic insults on human neuronal networks and, ultimately, to accelerate patient- and disease-specific therapy and drug discovery with a human-based assay.

## Materials and Methods

### CMOS-MEAs and acquisition system

We performed all electrical recordings using a custom-built acquisition platform based on components of the BioCam system (3Brain GmbH). We grew neuronal hippocampal cultures on CMOS-MEAs (BioChip 4096S+ from 3Brain GmbH) that integrate an array of 4096 recording electrodes (21 × 21 μm^2^ in size, 42 μm pitch) on an active area of (2.67 × 2.67 mm^2^) centered in a working area of (6 × 6 mm^2^). We used BrainWave software for data recording and spike detection (Supplementary Fig. S13) (all components were obtained from 3Brain GmbH).

### Preparation and coating of CMOS-MEAs

We sterilized CMOS-MEAs thoroughly outside the culture chamber ring using tissue moistened with EtOH 96%. We placed each chip into a sterile 100 × 20 mm Petri dish (Corning) and sterilized the entire culture chamber by filling it with 70% EtOH. After 20 minutes, we rinsed the CMOS-MEAs 4x with sterile double-distilled water (DDW) and dried it under a sterile laminar flow hood. Chips were pre-conditioned by overnight incubation at 37 °C and 5% CO_2_ with Complete Neurobasal Medium (CNM) containing 2% B-27 1% penicillin/streptomycin and 1% GlutaMax supplements (all reagents from Life Technologies). The next day, CNM was aspirated and chips were immediately coated with 50 μg/ml poly-DL-ornithine (PDLO) (Sigma-Aldrich) and incubated overnight at 37 °C and 5% CO_2_. We filled a 35 × 10 mm Petri dish (Corning) with sterile DDW and placed it inside the Petri dish beside the chip both to maintain appropriate humidity and to avoid evaporation of the coating reagents overnight. The next day, CMOS-MEAs were rinsed 4x with sterile DDW and left to dry under the hood before cell seeding.

### Preparation of primary rat hippocampal cultures

We obtained primary hippocampal neurons from brain tissues of embryos of Sprague-Dawley rats at day 18 (E18) according to a previously described method^57^. All work with primary cultures was performed in accordance with the Italian guidelines and regulations, and all animal procedures carried out in this work were approved by the institutional IIT Ethics Committee and by the Italian Ministry of Health and Animal Care (Authorization No. 110/2014-PR of the 19th of December 2014). Briefly, embryos were removed and decapitated and the brains were extracted from the skulls. We placed the brains in cold Hanks Balanced Salt Solution (HBSS) and after dissection hippocampi (typically 7–10 per preparation) were placed in 0.125% trypsin-EDTA and incubated for 30 minutes at 37 °C in a water bath to dissociate the tissue. We then blocked the trypsin activity by CNM, supplemented with 10% fetal bovine serum (FBS) and centrifuged the tubes for 5 minutes at 1200 rpm. The supernatant was discarded, and fresh CNM and 10% FBS were added. The hippocampi were dissociated gently by pipetting, and then the solution was filtered with a cell strainer and centrifuged for 7 minutes at 700 rpm. The supernatant was discarded, and cells were resuspended in CNM and counted using trypan blue and a hemocytometer. We seeded cells in 36 μl drops to reach the final density of 1500 cell/mm^2^ on the coated substrates and then incubated at 37 °C with 5% CO_2_ and 95% humidity. After 1.5 h, we added 1.6 ml of CNM to the culture chamber and incubated under the same conditions. For maintenance and cell culture growth, one-third of the medium was routinely replaced with a fresh CNM every four days. All reagents were obtained, unless indicated differently, from Life Technologies.

### Preparation of adult neural stem cells (NSCs)

All of our work with cultures was performed in accordance with the Italian guidelines and regulations, and adult rat hippocampal GFP-labeled NSCs were obtained from Millipore. These are ready-to-use primary cells derived from adult female Fisher rats, as described^58^, and are constitutively labeled with the humanized mulleri green fluorescence protein (hmGFP). We thawed the cells according to the manufacturer’s instructions and seeded them on a 10 cm tissue culture plate coated with 10 μg/ml poly-L-ornithine (PLO) and 5 μg/ml laminin. Cells were grown in an NSC basal medium containing 20 ng/ml FGF-2 and 1 μg/ml puromycin and incubated under standard conditions (37 °C with 5% CO_2_). We differentiated these multipotent NSCs after six passages into neurons using a neuron differentiation medium (NDM) containing 1 μM retinoic acid and 5 μM forskolin, and NDM was freshly replaced every other day for four days until 70% of the neuronal population was obtained. Following 80% confluency, NSCs were dissociated with Accutase, counted and seeded on the pre-existing network of hippocampal neurons at a final density of 1500 cell/mm^2^. All culturing medium and additional reagents were obtained from Millipore.

### Preparation of scrambled amyloid-β_1–42_ and amyloid-β_1–42_

Lyophilized peptides of Aβ_1–42_ and scrambled-Aβ_1–42_ were obtained from Eurogentec and resuspended at 1 mg/ml in 1,1,1,3,3,3-hexafluoro-2-propanol (HFIP) (Sigma-Aldrich) to obtain a homogeneous aggregate-free preparation as previously described^59^. We gently removed HFIP from samples with nitrogen stream, and stocks were stored at −80 °C until they were ready to use. We prepared fresh monomers and oligomers for each experiment. Prior to beginning the aggregation experiment, an optimized amount of peptide (25 μg) was fully resuspended in HFIP at 1 mg/ml, sonicated for 30 minutes in a water bath sonicator, dried, and resuspended at a concentration of 0.5 mg/ml in 10 mM phosphate buffer (PB) pH 7.4. Peptide aliquots were then sonicated for 10 minutes in a water bath sonicator at room temperature and centrifuged at 14,000 rpm for 30 minutes. The monomer-containing supernatant was used for further oligomerization. To obtain oligomers, 150 mM NaCl (Sigma-Aldrich) was added to the monomeric samples and incubated at 37 °C for 6 days. All preparation steps were performed using ultratrace metal-free reagents and water (Sigma-Aldrich).

### Western blotting

Monomers and oligomers Aβ_1–42_ were diluted from 0.5 µg in tricine-sodium dodecyl sulfate (SDS) buffer (Invitrogen) and electrophoresed on 10–20% SDS-tricine page gel (Invitrogen) for 90 minutes at 120 V. Subsequently, the proteins were transferred onto 0.2 µm polyvinylidene difluoride (PVDF) Immobilon P membranes (Millipore). The membranes were boiled for 10 minutes in phosphate-buffered saline (PBS) (Life Technologies); blocked with 5% nonfat dry milk in 20 mM Tris-HCl, pH 7.5, 500 mM NaCl, and 0.01% Tween 20; and probed with 6E10 or 4G8 anti-Aβ mouse monoclonal antibodies (R&D Systems; dilution 1:1000). This was followed by horseradish peroxidase-conjugated secondary antibodies (Bio-Rad). Immunoreactive bands were detected using a Western light chemiluminescence detection system (ECL, GE Healthcare Bio-Sciences AB) and then exposed and photographed in an ImageQuant LAS 4000 mini (GE Healthcare BioScience AB).

### Neuronal viability and toxicity assays

Two methods were used to quantify cell viability. First, neuronal viability was evaluated using 3-(4,5-dimethylthiozolyl)-2,5-diphenyl-tetrazolium bromide (MTT) (Sigma-Aldrich) that was developed for a 96-well format and suitable for high-throughput screening (HTC)^60^. A concentration of 1 mg/ml MTT substrate was prepared in a phosphate-buffered saline (PBS) and added to culture medium as 10%. Cells were incubated at 37 °C for 90 minutes until they turned blue. Cells were lysed in 5% Triton X-100 in 0.1 M Tris buffer pH 7.5 for 2 h in agitation. Fully lysed cells were examined under a bright field microscope indicated by complete detached cells. The conversion of MTT from a yellow to a blue formazan crystal by dehydrogenase enzymes in metabolically active cells was measured by detecting the changes in absorbance at 570 nm using a VICTOR^3^ V plate reader (PerkinElmer). Second, neuronal viability was evaluated using the active form of caspase-3 through immunofluorescence readouts, as described in the next section.

### Immunofluorescence protocol

We performed immunostaining on rat hippocampal cultures grown on CMOS-MEAs, glass coverslips and plastic multi-wells and microplates using a previously described protocol^27^. Briefly, we removed the culture medium and washed using phosphate-buffered saline (PBS-1X) (Life Technologies) at 37 °C, and then we fixed samples in paraformaldehyde (4% in PBS-1X) for 15 minutes at room temperature (RT). We washed the sample 4× with PBS-1X, then cells were permeabilized with 0.1% Triton X-100 in PBS-1X (PBST) for 10 minutes, and then cells were blocked with 5% normal goat serum (NGS) (EuroClone) for 1 h before incubation with primary antibodies. Cells were then incubated overnight at 4 °C in primary antibodies diluted in the NGS blocking buffer. The following primary antibodies were used: guinea pig anti-MAP2 (Synaptic Systems; 1:1000), rabbit anti-c-Fos (Calbiochem Millipore; 1:500), and rabbit anti-caspase-3 (BD Bioscience; 1:500). We performed 3 progressive washing steps in PBST for five minutes per wash, and cells were incubated for 1 h in the dark at RT with the corresponding secondary antibodies, including Alexa Fluor 488, Alexa Fluor 546, and Alexa Fluor 647 (all from Life Technologies; 1:1000). Subsequently, the cells were washed 4× with PBST and incubated for 15 minutes in the dark at RT with the nuclear marker Hoechst 33342 (Thermo Fisher Scientific; 1:500) diluted in PBS-1X. We visualized and acquired all images with 20x and 40x objective lenses using a Leica SP5 upright confocal microscope (Leica Microsystems), a Nikon A1 inverted confocal microscope (Nikon Instruments), a BX61 Olympus fluorescence microscope (Olympus Life Science Solutions), and BD Pathway 855 (BD Bioscience).

### Drug treatments

#### Memantine hydrochloride

We freshly prepared memantine hydrochloride for each experiment by dissolving 1 mg/ml (Sigma-Aldrich) in DDW. The final working solution used for all experiments was at a concentration of 10 µM. This concentration was co-administered either with Aβ-oligomers or after 26 h by pipetting it directly in the cell culture medium of CMOS-MEAs.

#### Stigmas of saffron (L’Aquila saffron)

To avoid using saffron from different cultivars that may present different compositions of the extract, we used only saffron derived from Hortus Novus (L’Aquila, Italy), whose chemical characteristics have been analytically examined in previous studies. This compound was freshly prepared for each experiment by soaking 5 mg/ml saffron in DDW and incubating it at 4 °C for 24 h (protected from light). The final working concentration used for all experiments was 25 µg/ml. This concentration was either co-administered with Aβ-oligomers or after 26 h by pipetting it directly to the cell culture medium.

#### NMDA

We prepared a fresh solution for each experiment by dissolving 1 mg/ml (Sigma-Aldrich) in DDW. Experiments were carried out in a dose-dependent protocol with increasing concentrations of 0.1, 0.5, 1, 5, 10, 30, 50, and 100 µM. Solution was added by pipetting it directly into the cell culture medium.

### Extracellular recordings and analyses of spiking activity

We performed multiple extracellular recordings on hippocampal neuronal networks; each session lasted 10 minutes. These recordings were acquired at a full-frame resolution of 7.8 KHz/electrode and from 4096 electrodes (at an acquisition rate of 60 Mbyte/s). The time stamps of the spikes were detected using the Brainwave software application (3Brain GmbH) and Precise Timing Spike Detection (PTSD). As in previous works, we adopted a threshold of 9× the standard deviation for spike detection, whereas burst events were identified if we detected at least five consecutive spikes in an inter-spike interval (ISI) lower than 100 ms. Only electrodes that recorded spikes with rates between 0.05 and 10 events/sec (active electrodes) were considered in our analysis. Given the electrode array density, the large majority of these electrodes recorded spikes from single units (>99%, spike sorting tests performed with the Plexon Offline sorter), which are therefore referred as putative single neurons. All spike trains were exported from BrainWave software in a MATLAB file format and were analyzed using custom Python scripts (Python Software Foundation). The analysis included first-order statistics of network-wide mean activity parameters (mean firing rate, mean bursting rate, and number of active electrodes) and an analysis of the neuronal firing frequency distributions. For the latter, lognormal-like distributions and fit statistics were analyzed as previously described^27^. Briefly, we assigned each active electrode to its corresponding averaged MFR value, then we normalized the occurrence of these active electrodes as proportion of their corresponding spiking activity divided by the total number of electrodes in the array, which is 4096. To determine changes in the spiking activity of single units, we performed pairwise Kolmogorov-Smirnov tests on the ISI distributions of the recorded phases before and after treatments or between baseline and end-point recordings, followed by Bonferroni’s correction on the replicates. We mainly detected significant differences that either arose from changes in spiking frequencies, as indicated by shorter or longer ISIs, or from spikes that were differently arranged in time, as indicated by higher or lower bursting activity, albeit with the same number of spikes. Accordingly, three groups of single firing units were classified based on the statistical significance of the changes in their mean firing rates, namely, increased, unchanged, and decreased unit classes.

### Image analyses

#### c-fos

To quantify the neuronal activity through cellular c-fos expression, we grew neuronal cultures on three different cell culture platforms. First, neuronal networks grown on CMOS-MEAs were acquired on an Leica SP5 upright microscope using a 25x/0.95 NA objective (Leica Microsystems). Second, cultures grown on glass coverslips were acquired on a Nikon A1 inverted microscope using a 20x/0.75 NA objective (Nikon Instruments). Third, to consolidate the cellular readouts of c-fos expression in high-throughput experimentation, we performed multiplexed high-content imaging (HCI) by growing neurons in 96 microplates (Corning). In this latter case, images were acquired on the BD pathway 855 Bioimager in the non-confocal mode using 20x/0.75 NA objective (BD Biosciences). Consequently, the quantification of c-fos expression for all these data was performed by counting the c-fos^+^ nuclei. Images acquired from CMOS-MEAs and coverslips were quantified using the object count tool of the NIS Element Advance Research Software AR (Nikon), which allowed us to perform automated object detection and counting by using image thresholding based on area, circularity, and EqDiameter parameters (Nikon NIS-Elements Advanced Research User’s Guide V.4). Images were acquired from at least 9 fields in each confocal image from four different preparations and different animals. For data acquired with the HCI platform, we quantified the c-fos^+^ nuclei expression using BD AttoVision software v1.6 (BD Biosciences), which allowed us to apply the polygon segmentation methods to generate polygon-shaped ROIs from a single dye (c-fos and Hoechst for the nuclei); then, the total number of ROIs indicated the positive expression of c-fos with respect to their nuclei.

#### Caspase-3

We quantified the activation of caspase-3 as a hallmark of apoptosis after Aβ-treatment. Neurons were grown on three different platforms, as described for c-fos quantifications. We evaluated the nuclear intensity by quantifying the gray levels of multiple nuclei in at least 9 fields in each confocal image from a minimum of four different preparations. We used the ImageJ analysis software platform^61^ to quantify caspase-3-caused fluorescence intensity changes for cultures grown on CMOS-MEAs and coverslips. The level of activated caspase-3 expressed by cultures grown in 96 microplates (Corning) for the HCI platform was analyzed using BD AttoVision software v1.6 (BD Bioscience) by quantifying the gray level in the segmented polygon-shaped ROIs, which indicated positive nuclear intensities of caspase-3 activation in Aβ-treated samples compared to those in wild-type samples.

### Statistical analyses and experimental parameters

All data in this work are expressed as the mean ± standard error of the mean (SEM). Differences between groups were examined for statistical significance, where appropriate using one-way analysis of variance (ANOVA) or two-way ANOVA followed by Tukey’s post-hoc testing. A non-parametric Kruskal-Wallis method was used for data that were not normally distributed. A non-parametric Kolmogorov-Smirnov test was used to assess the differences between distributions. *p* < *0*.*05* was considered significant. The statistical tests used are depicted in the figure captions.

To ensure the consistency and reproducibility of our results, we conducted at least three repeated trials performed in different culture preparations and from at least three different animals. The number of samples used in each experiment is depicted in the figure legends.

## Electronic supplementary material

Supplementary figures and legends
