# Supplementary material for: High-resolution bioelectrical imaging of Aβ-induced network dysfunction on CMOS-MEAs for neurotoxicity and rescue studies
Source: Sci Rep. 2017 May 26;7:2460. doi: 10.1038/s41598-017-02635-x (PMC5446416; doi:10.1038/s41598-017-02635-x)
Supplement: Supplementary file 1 — Supplementary figures and legends [file 41598_2017_2635_MOESM1_ESM.pdf]

High-resolution bioelectrical imaging of Aβ-induced network dysfunction on CMOS-MEAs for neurotoxicity and rescue studies

Hayder Amin*[1], Thierry Nieus[1], Davide Lonardoni[1], Alessandro Maccione[1], and Luca Berdondini[1]

[1]Nets[3] Laboratory, Departement of Neuroscience & Brain Technologies (NBT), Fondazione Istituto Italiano di Tecnologia (IIT), via morego 30, 16163 Genova, Italy

*Correspondence to: hayder.amin@iit.it

SUPPLEMENTARY FIGURES AND LEGENDS

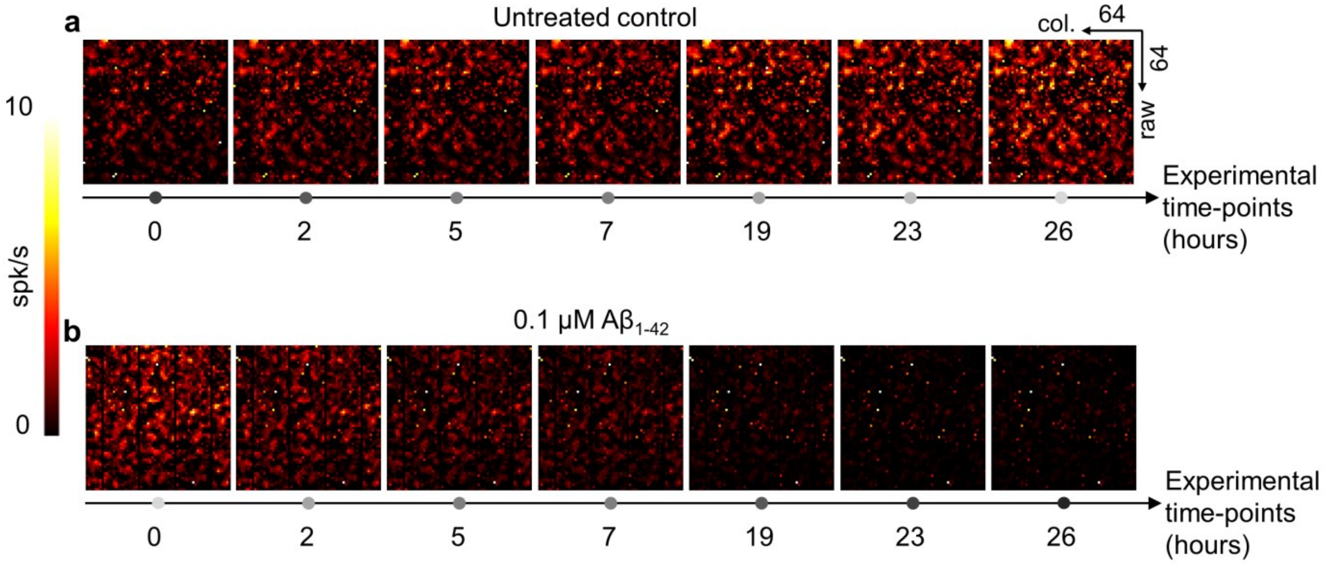

Figure S1 | Monitor at single-neuron resolution the dysregulation of neuronal network activity induced by Aβ-toxicity with electrical readouts from 4096-electrode arrays.

(a) Representative snapshots of the averaged MFR maps (64 x 64 electrode-pixels) computed for each experimental phase (7 recorded phases, over 26 h) of an untreated network, where the activity is gradually increasing, as a "light-on" pattern. (b) As in (a), but when network is treated after t=0 with 0.1 μM Aβ. The snapshots of the averaged MFR maps indicate a homogenous and gradual decrease of the neuronal firing activity, as a "light-off" pattern.

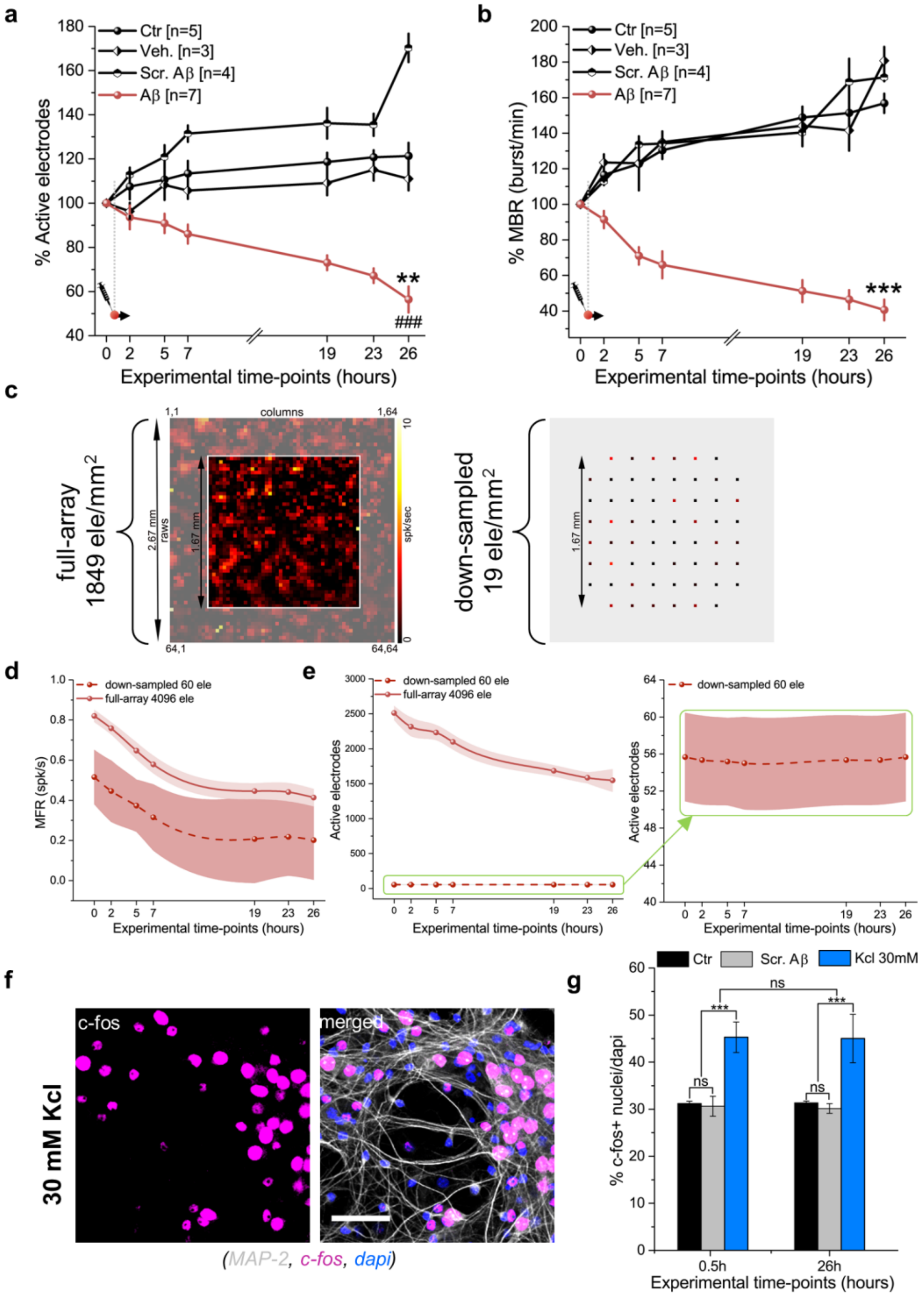

Figure S2 | Extended data from Fig. 2c-e.

(a) Number of active electrodes increases for control groups (black lines) and decreases after 26 h of exposure to 0.1 µM Aβ-oligomers (red line). ***Denotes $p < 0.01$* when compared to untreated ctr and veh, and ### denotes $p < 0.001$ when compared to scrambled-Aβ, ANOVA with Tukey's post hoc test. (b) Mean bursting rate (MBR) indicates a decreasing tendency of the network synchronization and its significant suppression after 26 h of Aβ-oligomers treatment (red line), with respect to control groups (black lines). ****$p < 0.001$*, ANOVA with Tukey's post hoc test. (c) Representative layouts of the full array (*left*) vs. the down-sampled network on 60 electrodes layout (*right*), to compare their averaged firing rates. (d) MFRs of Aβ-treated group plotted for the full-array vs. the down-sampled network recordings, where both are

significantly different over 7 phases of recordings. *p < 0.001*, Kolmogorov-Smirnov test. Down-sampled recordings show higher variability compared to full-array recordings. (e) Number of active electrodes of Aβ-treated group plotted for the full-array vs. the down-sampled network recordings, where both are significantly different over 7 phases of recordings. *p < 0.001*, Kolmogorov-Smirnov test. Down-sampled recordings show high-variability (*zoomed in; right*) and preclude the characterization of active-electrodes trend upon Aβ-treatment. (f) Representative confocal micrographs of a neuronal network showing the expression of cellular c-fos after 26 h of treatment with a positive control compound (30 mM KCl). Scale bar represents 50 µm. (g) Quantification of c-fos$^+$nuclei ratio showing a significant increase in c-fos expression after 0.5 h and 26 h of 30 mM Kcl compared to control and scrambled-Aβ groups, thus confirming the Aβ-oligomers induced decrease in c-fos expression illustrated in Fig. 2c. ****p < 0.001*, ANOVA with Tukey's post hoc test.

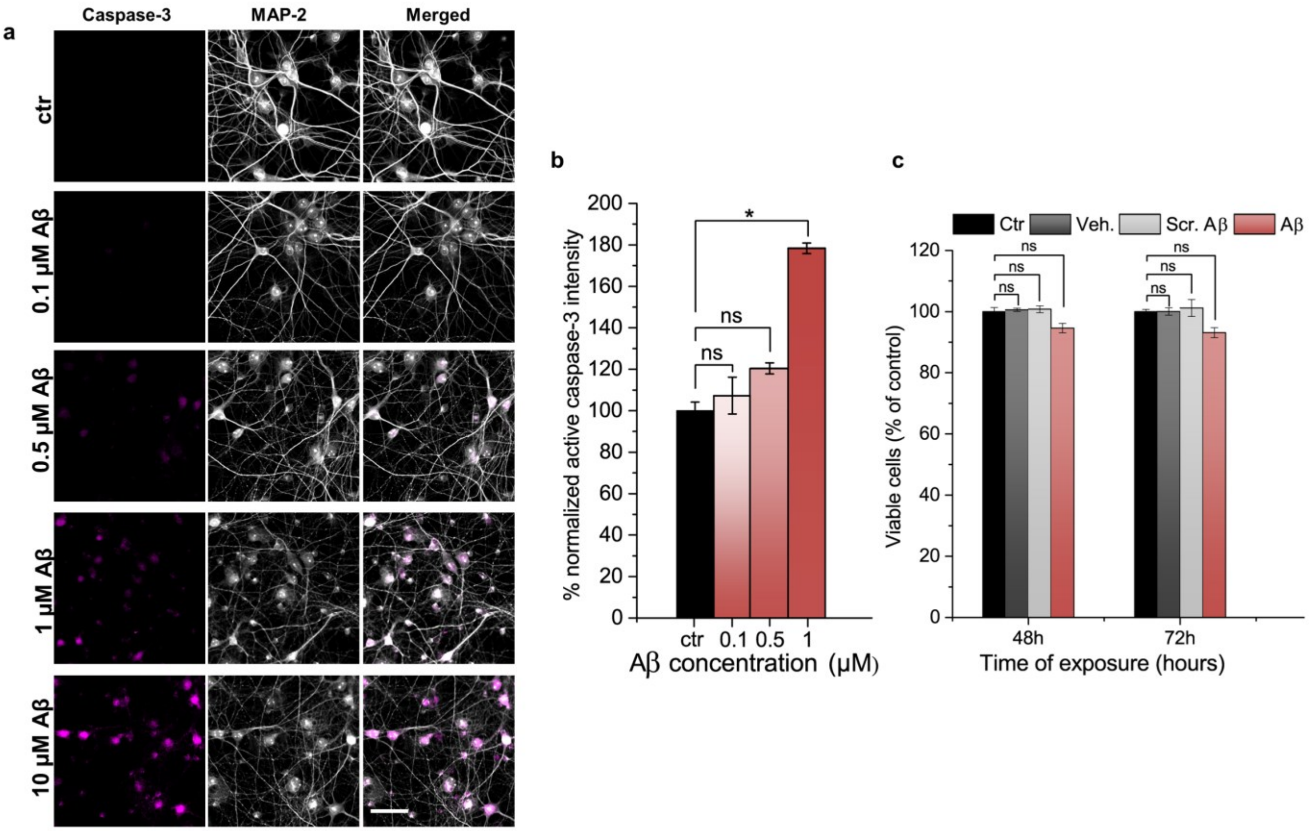

Figure S3 | Neuronal viability and toxicity validation extended over longer time and higher concentrations of Aβ-exposure.

(a) Representative confocal micrographs of hippocampal networks after 26 h showing Aβ-concentration-dependent caspase-3 activation as quantified in Fig. 2d. Scale bar represents 50 µm. (b) Intensity quantification of caspase-3 activity after 48 h of Aβ-increased concentration shows no apoptotic activation of hippocampal neurons at 0.1 µM, rather it is significantly present at 1 µM. **p < 0.05*, ANOVA. (c) Extended data of MTT-read-outs from Fig. 2e indicating no significant cell-death after 48 and 72 h of 0.1 µM Aβ-exposure.

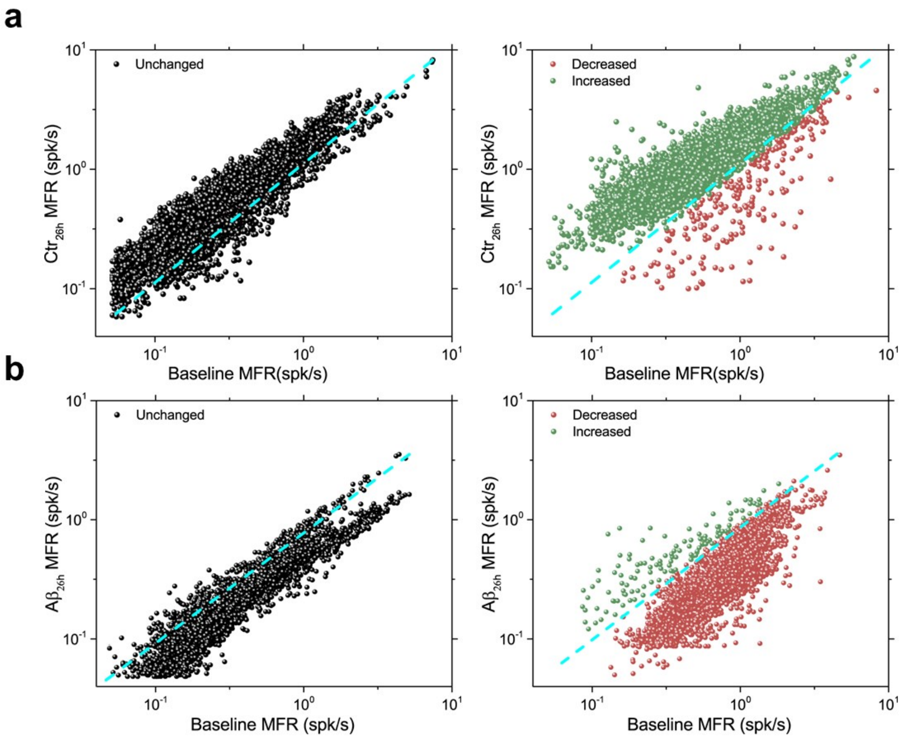

Figure S4 | Dot plots of all single active units as quantified in Fig. 2h, monitored for 26 h and classified in those showing unchanged, increased, decreased of the firing rates and confirming network dysfunction upon 26 h Aβ-exposure.

(a) Dot plots for changes in an untreated group, between baseline and after 26 h (Ctr). Single units are classified as described in (the methods section) to (left) unchanged, (right, green) increased, (right, red) decreased. (b) As in (a), but for changes in a treated group with 0.1 µM Aβ-oligomers (Aβ). The treated group is predominated by the decreased firing units (red) compared to the unchanged units in the control group.

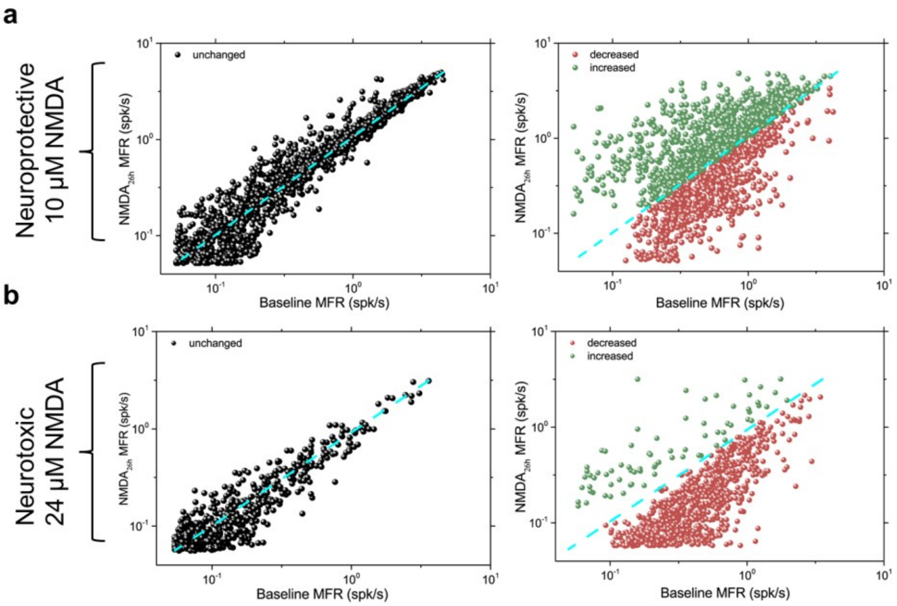

Figure S5 | Dot plots of all single active units as quantified in Fig. 3e, monitored for 26 h and classified in those showing unchanged, increased, decreased of the firing rates upon exposure with neuroprotective and neurotoxic NMDA doses.

(a) Dot plots for changes in a group treated with 10 µM NMDA, between baseline and after 26 h of exposure (neuroprotective NMDA dose). Single units are classified as described in (the methods section) to (left) unchanged, (right, green) increased, (right, red) decreased. (b) As in (a), but for changes in a group treated with 24.5 µM NMDA (neurotoxic NMDA dose). The group treated with a neurotoxic NMDA dose is predominated by the decreased firing units (red) compared to the unchanged units in the neuroprotective group.

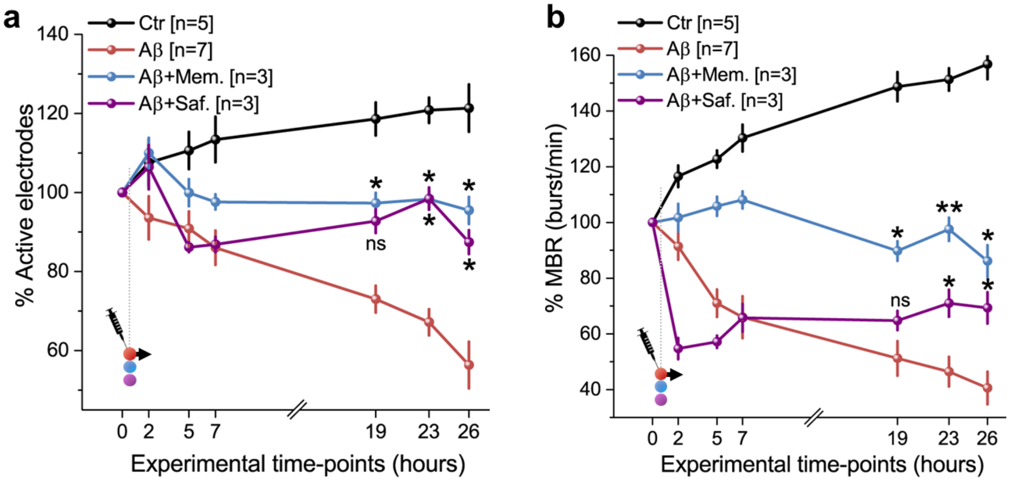

Figure S6 | Extended data from Fig. 4b.

Network-wide activity responses evaluated in terms of number of active electrodes and mean bursting rate (MBR) for two tested compounds co-administrated with Aβ-oligomers.

(a) Memantine at 10 µM and saffron at 25 µg/ml both show a potential neuroprotective effect by preserving the number of firing electrodes compared to Aβ-treated cultures. *$p < 0.05$*, ANOVA with Tukey's post hoc test. ns denotes "not significant when compared to the Aβ-treated group". (b) Both memantine and saffron significantly maintain the network synchronization as indicated by the MBR compared to Aβ treated networks. *$p < 0.05$*, ANOVA with Tukey's post hoc test. ns denotes "not significant when compared to the Aβ-treated group".

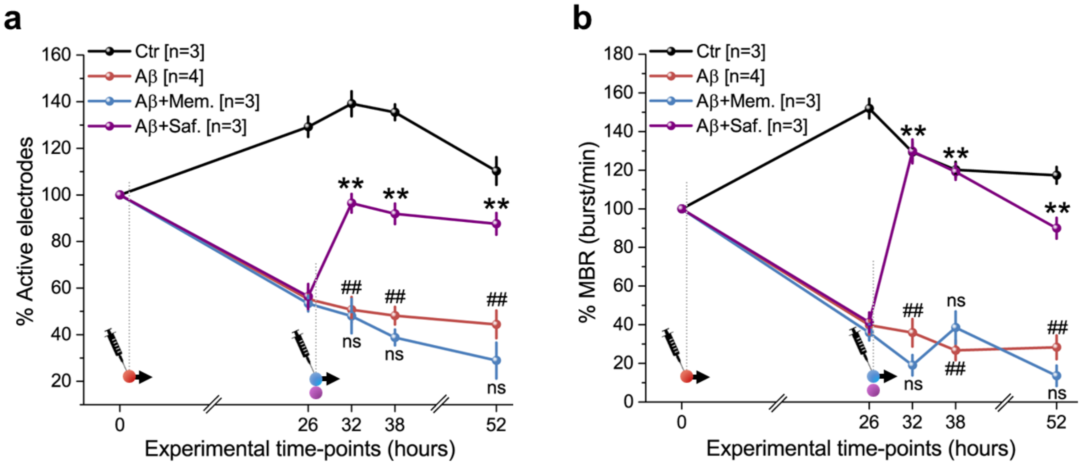

Figure S7 | Extended data from Fig. 4f.

Network-wide activity responses evaluated in terms of number of active electrodes and mean bursting rate (MBR) for two tested compounds administrated 26 h after Aβ-oligomers induced neurodegeneration.

(a) Saffron at 25 µg/ml shows a potent effect over 52 h to rescue the dysfunction of the neuronal network by preserving the number of firing electrodes compared to Aβ-treated cultures (0.1 µM), while when the degeneration is in-place, no significant rescuing effect is observed with memantine treatment. $^{*}p < 0.05$, ANOVA with Tukey's post hoc test. ns denotes "not significant when compared to the Aβ-treated group". (b) Similarly, only saffron shows a potent effect over 52 h to rescue the network synchronization properties as indicated by the MBR compared to the Aβ-treated group. ** Denotes $p < 0.01$ when compared to Aβ*,* and ## denotes $p < 0.01$ when compared to control, ANOVA with Tukey's post hoc test. ns denotes "not significant when compared to the Aβ-treated group ".

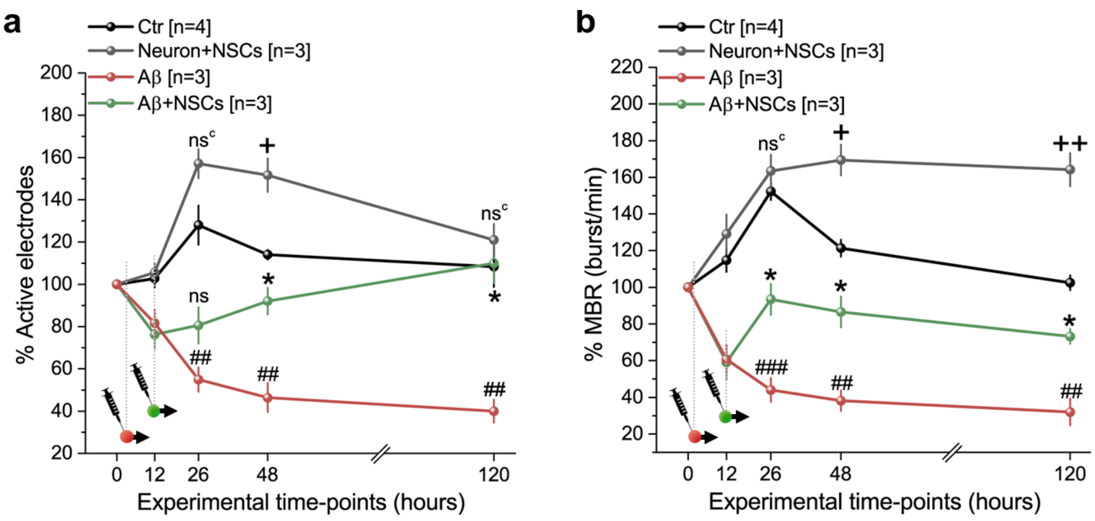

Figure S8 | Extended data from Fig. 5c.

Network-wide activity responses evaluated in terms of number of active electrodes and mean bursting rate (MBR) upon cell-therapy using NSCs administrated 12 h after Aβ-oligomers induced neurodegeneration.

(a) NSCs integrating into pre-existing networks treated with 0.1 µM of Aβ-oligomers show a significant potent rescuing effect on the number of firing electrodes compared to cultures treated only with Aβ. * Denotes $p < 0.05$ when compared to Aβ. ## Denotes $p < 0.01$ and + denotes $p < 0.05$ when compared to control, ANOVA with Tukey's post hoc test. ns denotes "not significant when compared to the Aβ-treated group", and ns[c] denotes "not significant when compared to control". (b) In the same way, NSCs appear to rescue the network synchronization, as indicated by the MBR compared to cultures treated only with Aβ-oligomers. * Denotes $p < 0.05$ when compared to Aβ. ## Denotes $p < 0.01$, + denotes $p < 0.05$, and ++ denotes $p < 0.01$ when compared to control, ANOVA with Tukey's post hoc test. ns[c] denotes "not significant when compared to control".

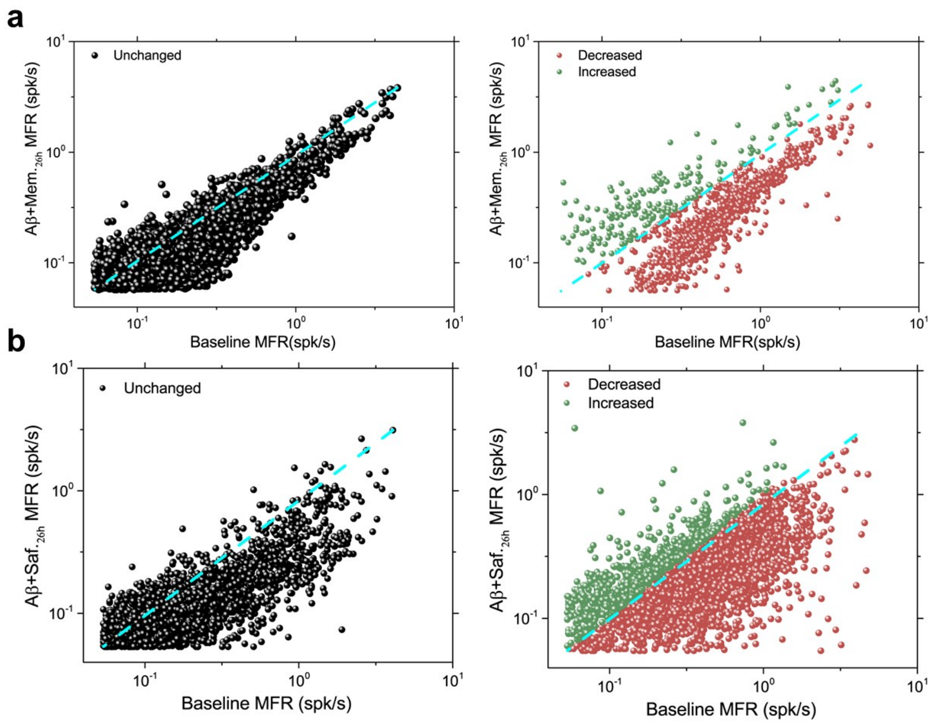

Figure S9 │ Dot plots of all single active units as quantified in Fig. 4d, monitored for 26 h and classified in those showing unchanged, increased, decreased of the firing rates upon treatment with compounds co-administrated with Aβ-oligomers.

(a) Dot plots for changes in the group treated with co-administered Aβ+memantine, between baseline and after 26 h. Single units are classified as described in (the methods section) to (left) unchanged, (right, green) increased, (right, red) decreased. (b) As in (a), but for changes in a group treated with co-administered Aβ+saffron, between baseline and after 26 h of exposure.

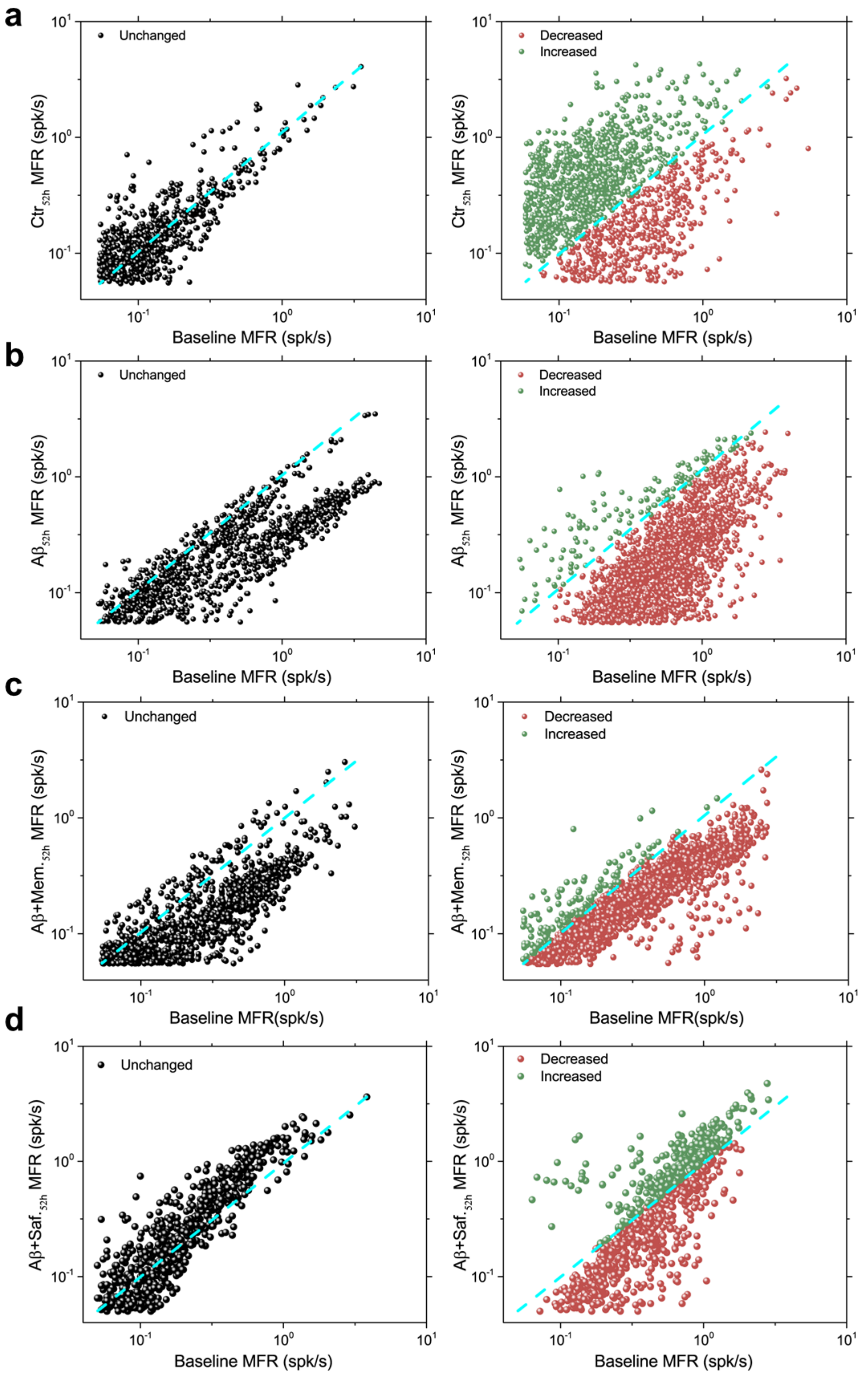

Figure S10 │ Dot plots of all single active units as quantified in Fig. 4h, monitored for 52 h and classified in those showing unchanged, increased, decreased of the firing rates upon treatment with compounds administrated 26 h after Aβ-oligomers induced neurodegeneration.

(a) Dot plots for changes in an untreated group, between baseline and after 52 h (Ctr). Single units are classified (see the methods section) to (left) unchanged, (right, green) increased, (right, red) decreased. (b) As in (a), but for changes in a group treated with 0.1 µM Aβ-oligomers (Aβ). (c) As in (a), but for changes in the group treated with memantine (Mem. after Aβ). (d) As in (a), but for changes in the group treated with saffron (Saf. after Aβ).

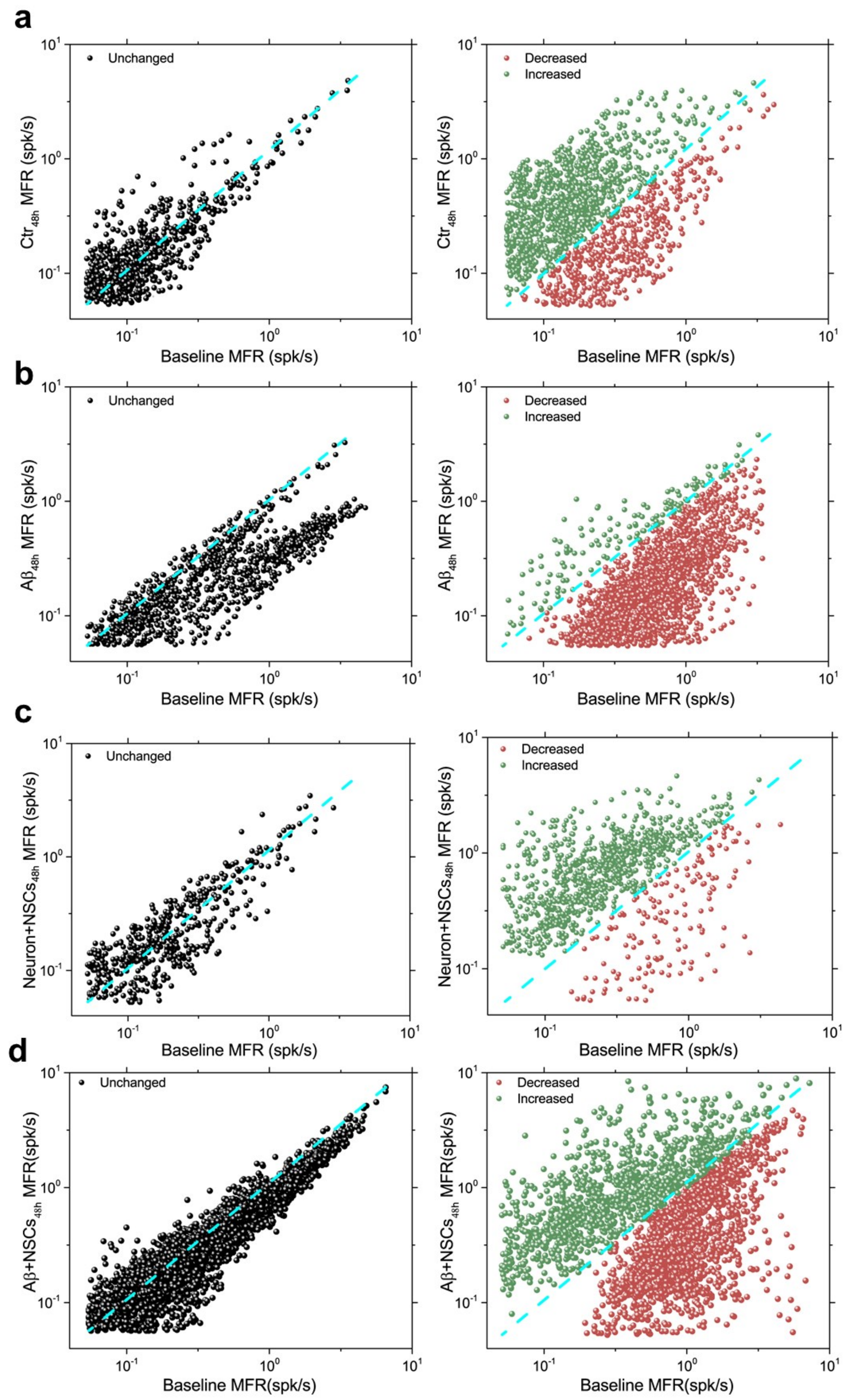

Figure S11 | Dot plots of all single active units as quantified in Fig. 5e, monitored for 48 h and classified in those showing unchanged, increased, decreased of the firing rates upon NSCs-therapy at 12 h after Aβ-induced toxicity.

(a) Dot plots for changes in an untreated group, between baseline and after 48 h (Ctr). Single units are classified as described in (the methods section) to (left) unchanged, (right, green) increased, (right, red) decreased. (b) As in (a), but for changes in a group treated with 0.1 μM Aβ-oligomers (Aβ). (c) As in (a), but for changes in healthy networks where NSCs were added (Neuron+NSCs). (d) As in (a), but for changes in the group where NSCs added to a diseased pre-existing networks, 12 h after toxicity was induced by Aβ-oligomers (Aβ+NSCs).

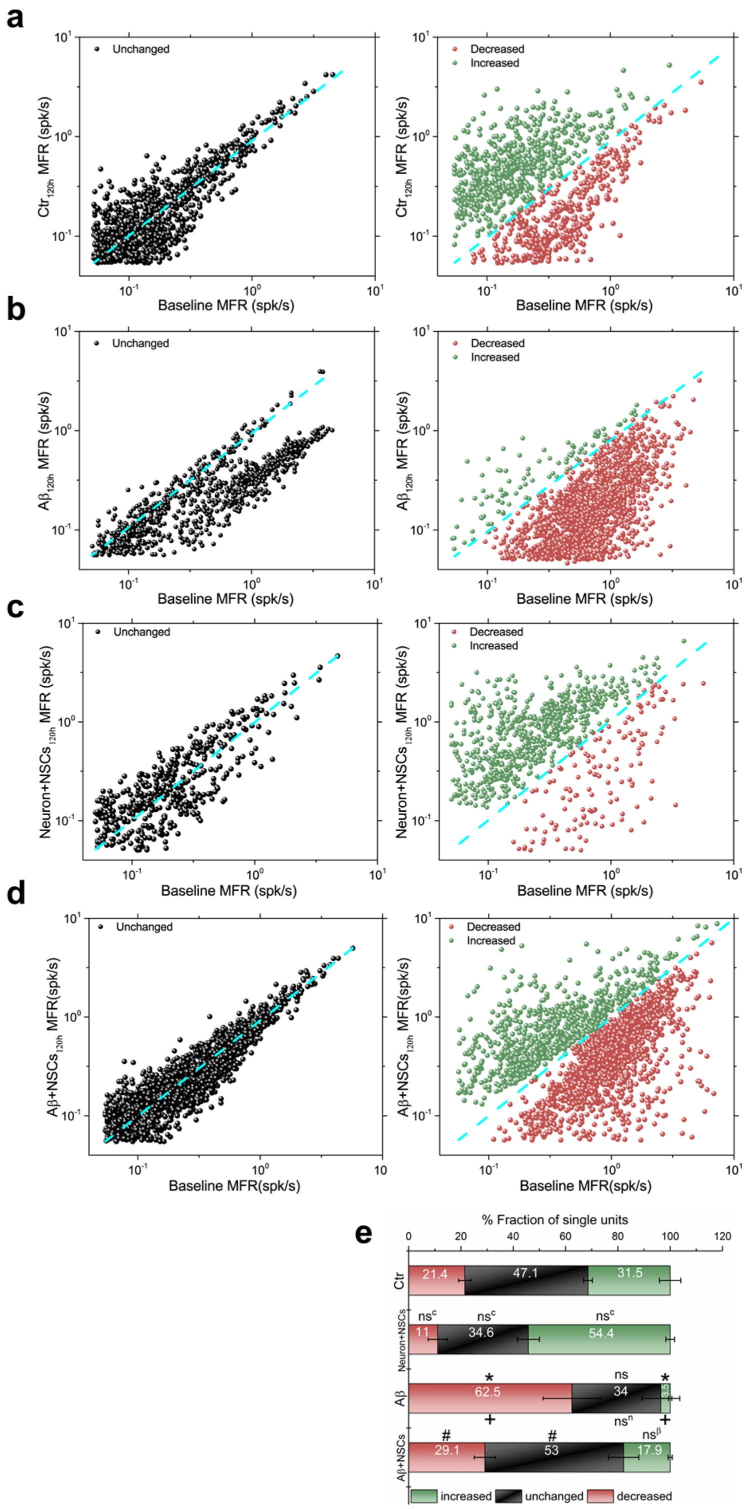

Figure S12 │ Dot plots and quantification of all single active units monitored for 120 h and classified in those showing unchanged, increased, decreased of the firing rates upon NSCs-therapy at 12 h after Aβ-induced toxicity.

(a) Dot plots for changes in an untreated group, between baseline and after 120 h (Ctr). Single units are classified as described in (the methods section) to (left) unchanged, (right, green) increased, (right, red) decreased. (b) As in (a), but for changes in a group treated with 0.1 µM Aβ-oligomers (Aβ). (c) As in (a), but for changes in the group, where NSCs added to

healthy networks (Neuron+NSCs). (d) As in (a), but for changes in the group, where NSCs added to a diseased pre-existing networks, 12 h after toxicity was induced by Aβ-oligomers (Aβ+NSCs). (e) Quantification of data reported in (a-d). * and + denote $p < 0.05$ when compared to control and Neuron+NSCs, respectively. # Denotes $p < 0.05$ when compared to Aβ, ANOVA with Tukey's post hoc test. ns, ns^{β}, and ns^{c} denote not significant when compared to control, Neuron+NSCs, and Aβ, respectively.

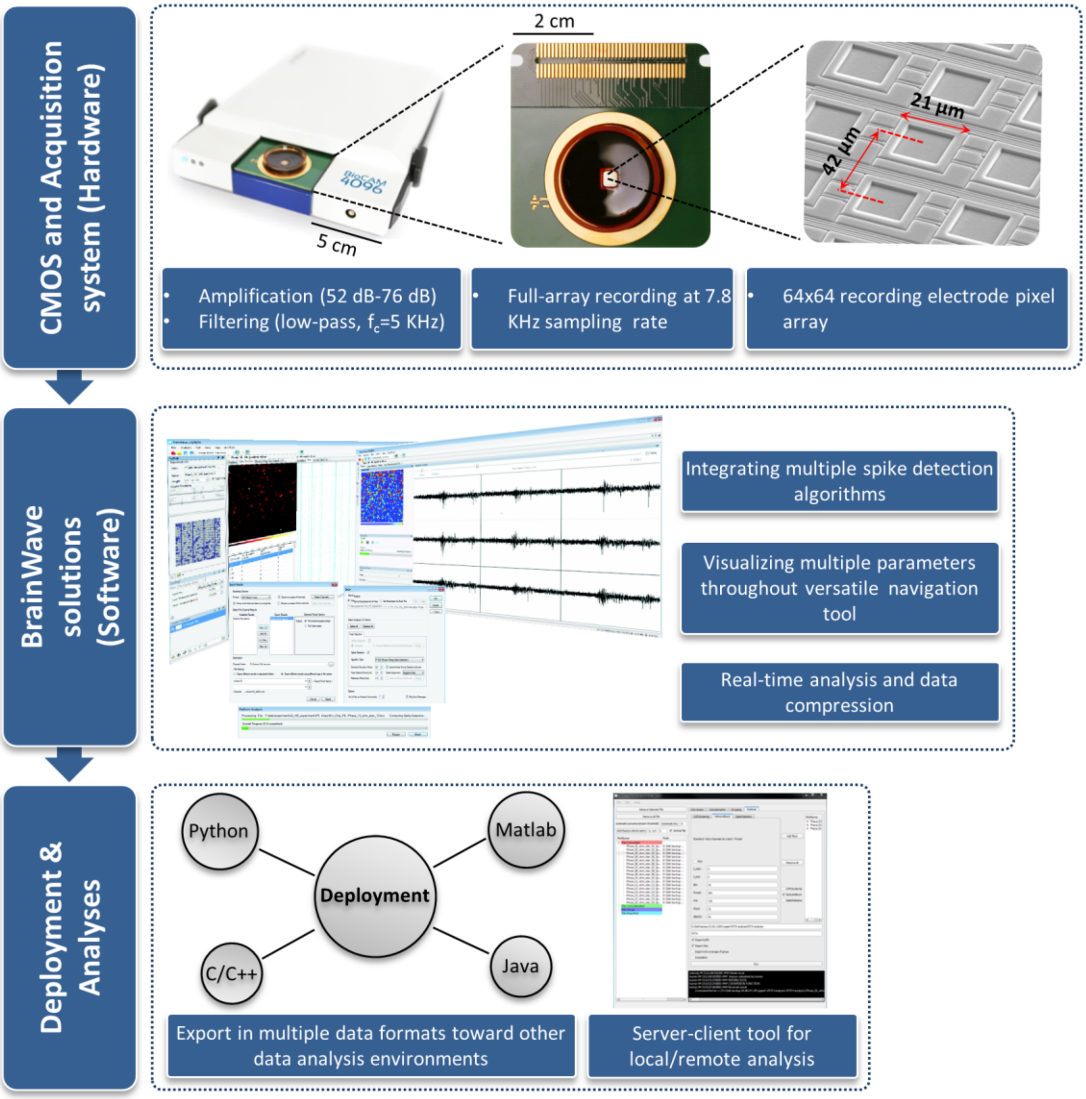

Figure S13 | Schematic of the CMOS-MEA platform and the acquisition system.
